# Harnessing Few-Shot Learning for EEG signal classification: a survey of state-of-the-art techniques and future directions

**DOI:** 10.3389/fnhum.2024.1421922

**Published:** 2024-07-10

**Authors:** Chirag Ahuja, Divyashikha Sethia

**Affiliations:** ^1^Department of Computer Science and Engineering, Delhi Technological University, New Delhi, India; ^2^Department of Software Engineering, Delhi Technology University, New Delhi, India

**Keywords:** EEG signals, Few-Shot Learning, Data Augmentation, Transfer Learning, Auto Augment, Subject Invariance, spatiotemporal modeling, geometric transformations

## Abstract

This paper presents a systematic literature review, providing a comprehensive taxonomy of Data Augmentation (DA), Transfer Learning (TL), and Self-Supervised Learning (SSL) techniques within the context of Few-Shot Learning (FSL) for EEG signal classification. EEG signals have shown significant potential in various paradigms, including Motor Imagery, Emotion Recognition, Visual Evoked Potentials, Steady-State Visually Evoked Potentials, Rapid Serial Visual Presentation, Event-Related Potentials, and Mental Workload. However, challenges such as limited labeled data, noise, and inter/intra-subject variability have impeded the effectiveness of traditional machine learning (ML) and deep learning (DL) models. This review methodically explores how FSL approaches, incorporating DA, TL, and SSL, can address these challenges and enhance classification performance in specific EEG paradigms. It also delves into the open research challenges related to these techniques in EEG signal classification. Specifically, the review examines the identification of DA strategies tailored to various EEG paradigms, the creation of TL architectures for efficient knowledge transfer, and the formulation of SSL methods for unsupervised representation learning from EEG data. Addressing these challenges is crucial for enhancing the efficacy and robustness of FSL-based EEG signal classification. By presenting a structured taxonomy of FSL techniques and discussing the associated research challenges, this systematic review offers valuable insights for future investigations in EEG signal classification. The findings aim to guide and inspire researchers, promoting advancements in applying FSL methodologies for improved EEG signal analysis and classification in real-world settings.

## 1 Introduction

Electroencephalography (EEG) is a non-invasive neuroimaging technique that measures electrical activity in the brain. It has been extensively used in research and clinical settings to study brain function and diagnose neurological disorders. However, the analysis of EEG signals presents significant challenges due to the low signal-to-noise ratio, high dimensionality, and inter-individual variability in EEG features (Rashid et al., 2020; Alvi et al., 2022). Recently, a machine learning method called Few-Shot Learning (FSL) has become popular for improving EEG signal analysis. FSL is designed to work well even when only a small number of examples are available for training (Chen et al., 2019; Wang and Yao, 2019). This is particularly useful for EEG analysis, where collecting a lot of labeled data can be hard, expensive, and time-consuming. FSL techniques have shown that they can accurately classify and analyze EEG signals with only a few labeled samples (Bajaj et al., 2020; Dzedzickis et al., 2020; Lin et al., 2020). This makes FSL a powerful tool for EEG signal classification, helping to overcome some of the major challenges in the field. By using FSL, researchers can achieve accurate results without needing large amounts of labeled data, making it a valuable approach for EEG studies.

Furthermore, Few-Shot Learning (FSL) enhances the robustness of the model against the inherent inter-individual variability found in EEG features. By leveraging knowledge from prior tasks or datasets through transfer learning, FSL techniques can effectively identify and generalize patterns across individuals, thereby improving the performance of EEG signal classification models (Schonfeld et al., 2019; Zhuang et al., 2020). Despite its advantages, FSL encounters challenges in EEG analysis, such as limited labeled datasets, subject variations, and adaptability to new datasets (Song et al., 2022). However, recent advancements in FSL strategies, including Data Augmentation (DA), Transfer Learning (TL), and Self-Supervised Learning (SSL), exhibit promising potential in mitigating these challenges (Gidaris et al., 2019; Li et al., 2019b; Chen et al., 2021).

Having a robust model is an essential part of any Machine Learning modeling, but due to lack of data, less data diversity or overfitting leads to a poor performance of the unseen data, causing the model to be unstable and can change its predictions with slight changes in the input. All this brings to a need for a mechanism that can overcome these challenges. Data augmentation (DA) addresses these challenges of real-world problems by synthetically generating data near the real world and adding it to the training process. However, developing a robust model near the real world does not solve other practical problems, such as the evolving nature of the world yielding new unseen data characteristics and hence bringing a need to adapt the pretrained model to a new data domain; this gives rise to Transfer Learning (TL), which can transfer new knowledge acquired to the existing model without the need of training from scratch. Transfer Learning (TL) can work if the underlying model is robust and rich enough to understand the intricacies of the data; therefore, leveraging the large amount of unlabeled data becomes very important to learn a rich representation of the data to have a pre-trained model that then can be used for fine tuning, this technique is referred to as Self Supervised Learning (SSL). This paper explores the work done in these paradigms from the lens of achieving FSL.

This systematic review sets itself apart significantly from existing reviews that have delved into methodologies such as Data Augmentation (DA), Transfer Learning (TL), and Self-Supervised Learning (SSL) within the domain of EEG signal processing. Unlike previous reviews that predominantly focused on specific facets of these techniques, such as SSL in Rafiei et al. (2022), TL in Redacted (2021), and DA in He et al. (2021), this research takes a more comprehensive approach. The study introduces an innovative taxonomy integrating DA, TL and SSL, providing a holistic perspective on Few-Shot Learning (FSL) for EEG signal classification. This taxonomy meticulously categorizes and organizes these techniques, establishing a structured foundation for comprehending their applicability across a broad spectrum of EEG paradigms. Moreover, the review critically highlights the differences and shortcomings in existing research, offering insights into best practices for evaluating and selecting the most suitable FSL strategy.

This review transcends the scope of existing literature by actively examining each of these FSL techniques within the context of different EEG paradigms. Rather than focusing solely on the methodologies themselves, it investigates their performance and adaptability across a diverse spectrum of EEG paradigms, encompassing tasks such as Motor Imagery (MI), Emotion Recognition (ER), Visual Evoked Potentials (VEP), Steady-State Visually Evoked Potentials (SSVEP), Rapid Serial Visual Presentation (RSVP), Event-Related Potentials (ERP), and Mental Workload (MWD). By actively considering FSL methodologies alongside distinct EEG paradigms, this research not only bridges gaps in the existing literature but also lays the groundwork for a more comprehensive understanding of FSL's potential in EEG signal processing. This comprehensive perspective actively contributes to the academic discourse by providing a valuable reference that actively assists researchers in navigating and advancing the domain of EEG signal processing with FSL techniques. The field employs a variety of techniques and methodologies, each with its own set of acronyms and terminology. To facilitate understanding, Table 1 provides a comprehensive list of acronyms and their definitions used throughout this paper.

**Table 1:** Acronyms and definitions.

**Acronym**	**Definition**
EEG	Electroencephalography: A non-invasive method to measure electrical activity of the brain.
MI	Motor Imagery: Imagining movement without actual execution, used in brain-computer interfaces and neurorehabilitation.
ER	Emotion Recognition: Detecting and analyzing emotional states, used in psychological studies and affective computing.
VEP	Visual Evoked Potentials: Brain responses to visual stimuli, used in vision research and clinical assessments.
SSVEP	Steady-State Visually Evoked Potentials: Steady response to flickering visual stimuli, used in brain-computer interfaces and attention tracking.
RSVP	Rapid Serial Visual Presentation: Rapid sequential presentation of visual stimuli, used in cognitive processing and attention studies.
ERP	Event-Related Potentials: Brain responses triggered by specific events, used in cognitive neuroscience and clinical diagnostics.
MWD	Mental Workload Detection: Assessing cognitive workload during tasks, used in human factors and usability testing.
FSL	Few-Shot Learning: Learning from a limited number of examples to classify or regress unseen data with minimal labeled samples.
DA	Data Augmentation: Techniques to increase the amount of data by adding modified copies of already existing data or newly created synthetic data from existing data.
TL	Transfer Learning: Reusing a pre-trained model on a new, but related, task to improve learning efficiency and performance.
SSL	Self-Supervised Learning: Learning useful representations from unlabeled data by solving pretext tasks.
GAN	Generative Adversarial Network: A class of machine learning frameworks designed to generate new data samples that resemble a given dataset.
WGAN	Wasserstein GAN: A variant of GAN that uses the Earth-Mover distance to improve training stability and quality of generated samples.
cWGAN	Conditional Wasserstein GAN: A variant of WGAN that incorporates auxiliary labels to improve generative performance.
VAE	Variational Autoencoder: A type of autoencoder that generates data samples by learning a probability distribution over the latent space.
LOOCV	Leave-One-Subject-Out Cross-Validation: A cross-validation method where one subject is used as the validation set, and the remaining subjects are used for training.
BCI	Brain-Computer Interface: A system that enables direct communication between the brain and external devices.
CNN	Convolutional Neural Network: A class of deep neural networks commonly used in visual and spatial data processing.
SVM	Support Vector Machine: A supervised learning algorithm used for classification and regression tasks.
MAML	Model-Agnostic Meta-Learning: An optimization-based meta-learning algorithm designed to train models that can adapt quickly to new tasks with minimal data.

The contributions of this review paper are:

- **Comprehensive analysis of existing literature:** This paper rigorously examines the current literature on Few-Shot Learning (FSL) techniques tailored for EEG signal classification. Evaluating a diverse range of studies furnishes a detailed overview of the prevailing state-of-the-art FSL methodologies in EEG analysis.
- **Evaluation of FSL techniques:** The paper critically assesses the merits and limitations of various FSL techniques within EEG signal classification. It scrutinizes the strengths and pitfalls of DA, TL, and SSL methods, considering challenges like limited labeled data and inter-individual variability in EEG analysis.
- **Identification of key findings and trends:** Through an exhaustive literature analysis, this paper pinpoints key findings and emerging trends in applying FSL techniques to EEG signal classification. It underscores vital considerations, such as the influence of distinct EEG paradigms on the selection and efficacy of FSL methods.
- **Highlighting research gaps:** By analyzing existing literature, this paper illuminates essential research voids in the Few-Shot Learning (FSL) domain for EEG analysis. Instead of merely suggesting future directions, this study accentuates areas warranting deeper exploration. These gaps encompass addressing current limitations, innovating FSL strategies, and probing the amalgamation of diverse techniques to augment the precision and efficiency of EEG signal classification.
- **Proposed the best practices for conducting FSL research**: This paper points out a few best practices to conduct FSL research and guidelines on how future research should report their results for better reproducibility and clarity.

This review paper meticulously evaluates the extant literature on FSL techniques for EEG signal classification, gauges the effectiveness of different FSL methods, discerns pivotal findings and trends, and provides insights into prospective research avenues. These contributions aspire to enlighten researchers and steer further progress in employing FSL methodologies to refine EEG signal analysis and classification.

**Figure 1:**
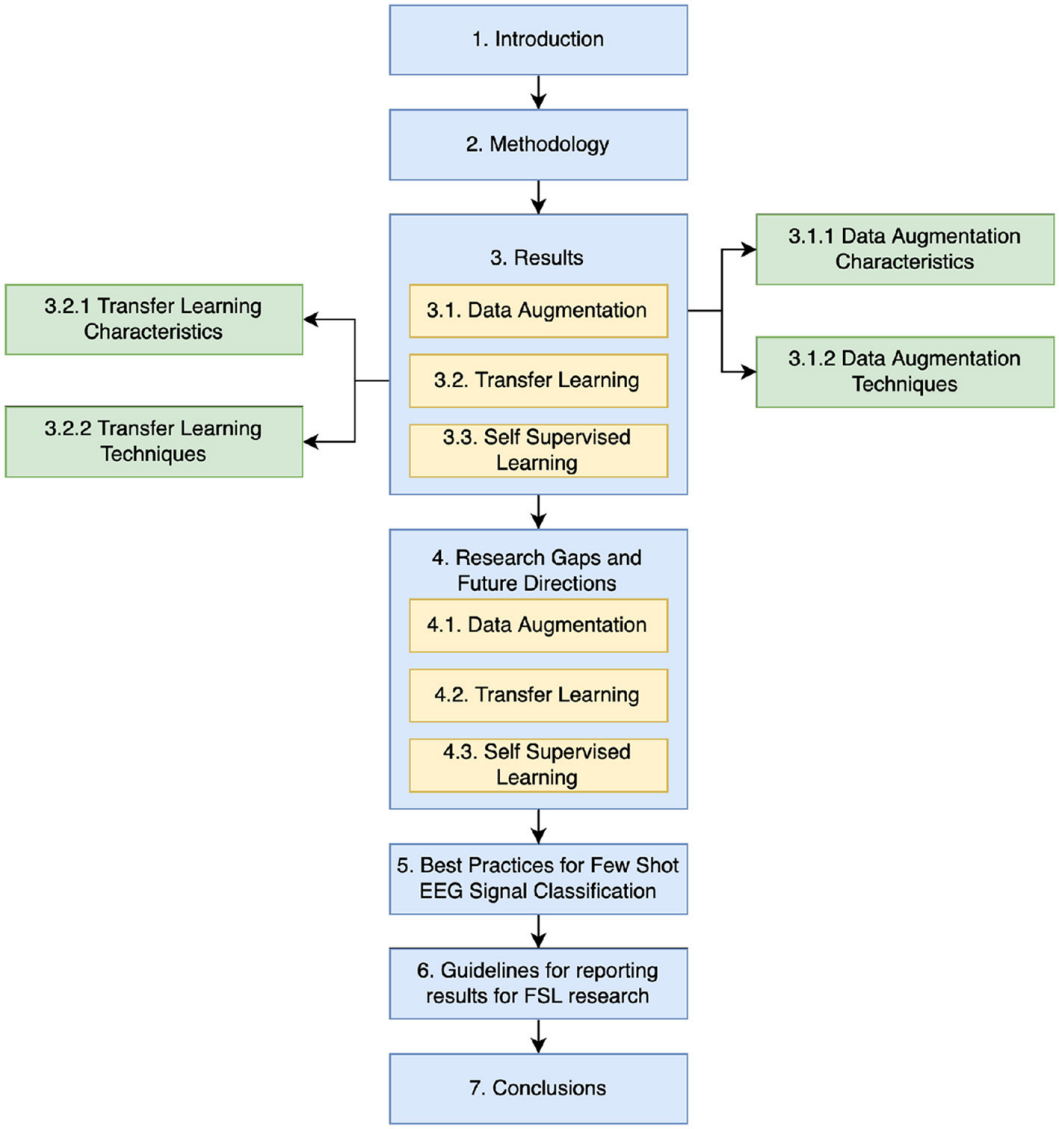
Structure of the paper.

The structure of this paper unfolds as follows as in Figure 1: Section 2 delineates the methodology adopted for this review. Section 3 unveils the review's findings and proposes a taxonomy grounded in the outcomes. Section 4 delves into a comprehensive discussion of the identified challenges. After reviewing previous literature, this paper layouts some Best Practices in Section 5 and proposes Guidelines for reporting results for future work in FSL in Section 6. Conclusively, Section 6 encapsulates the salient findings, contributions, prospective research trajectories, and the relevance of Few-Shot Learning (FSL) in EEG analysis.

## 2 Methodology

This review utilizes a systematic approach to ensure the search strategy's comprehensiveness and the results' accuracy. The search spans multiple databases, including PubMed, IEEE Xplore, and Google Scholar, targeting articles published between January 2015 and March 2023. The search keywords encompass “EEG” or “electroencephalography,” combined with terms related to “signal classification,” “pattern recognition,” and specific EEG paradigms. Additionally, terms associated with data augmentation techniques, such as “GAN,” “VAE,” and “Autoencoder,” are integrated as popular generative methods for synthesizing data to augment EEG datasets.

Boolean search strings are constructed to refine the search, including combinations such as [Data Augmentation AND (EEG OR electroencephalography)], [EEG AND (GAN OR VAE OR AutoEncoders)], [Transfer Learning AND (EEG OR electroencephalography)], and [Self Supervised AND (EEG OR electroencephalography)]. Table 2 presents the common EEG paradigms used in the query search in the review. There are other paradigms, too, as the initial search started with ER and MI and then, based on results, it is fixed to a few for the focus of this study. This approach aims to yield specific, easily aggregatable results. These queries yielded many duplicate records, which were filtered out by paper name. Also, as this work focuses on exploring the impact of Data Augmentation (DA), Transfer Learning (TL) and Self Supervised Learning (SSL) therefore, only the papers showcase the improvements with either of these techniques rather than modeling algorithms or hyperparameter tuning.

**Table 2:** EEG paradigms.

**Paradigm**	**Description**	**Applications**
Motor Imagery (MI)	Imagining movement without actual execution	Brain-computer interfaces, neurorehabilitation
Emotion Recognition (ER)	Detecting and analyzing emotional states	Psychological studies, affective computing
Visual Evoked Potentials (VEP)	Brain responses to visual stimuli	Vision research, clinical assessments
Steady-State Visually Evoked Potentials (SSVEP)	Steady response to flickering visual stimuli	Brain-computer interfaces, attention tracking
Rapid Serial Visual Presentation (RSVP)	Rapid sequential presentation of visual stimuli	Cognitive processing, attention studies
Event-Related Potentials (ERP)	Brain responses triggered by specific events	Cognitive neuroscience, clinical diagnostics
Mental Workload (MWD)	Assessing cognitive workload during tasks	Human factors, usability testing

**Figure 2:**
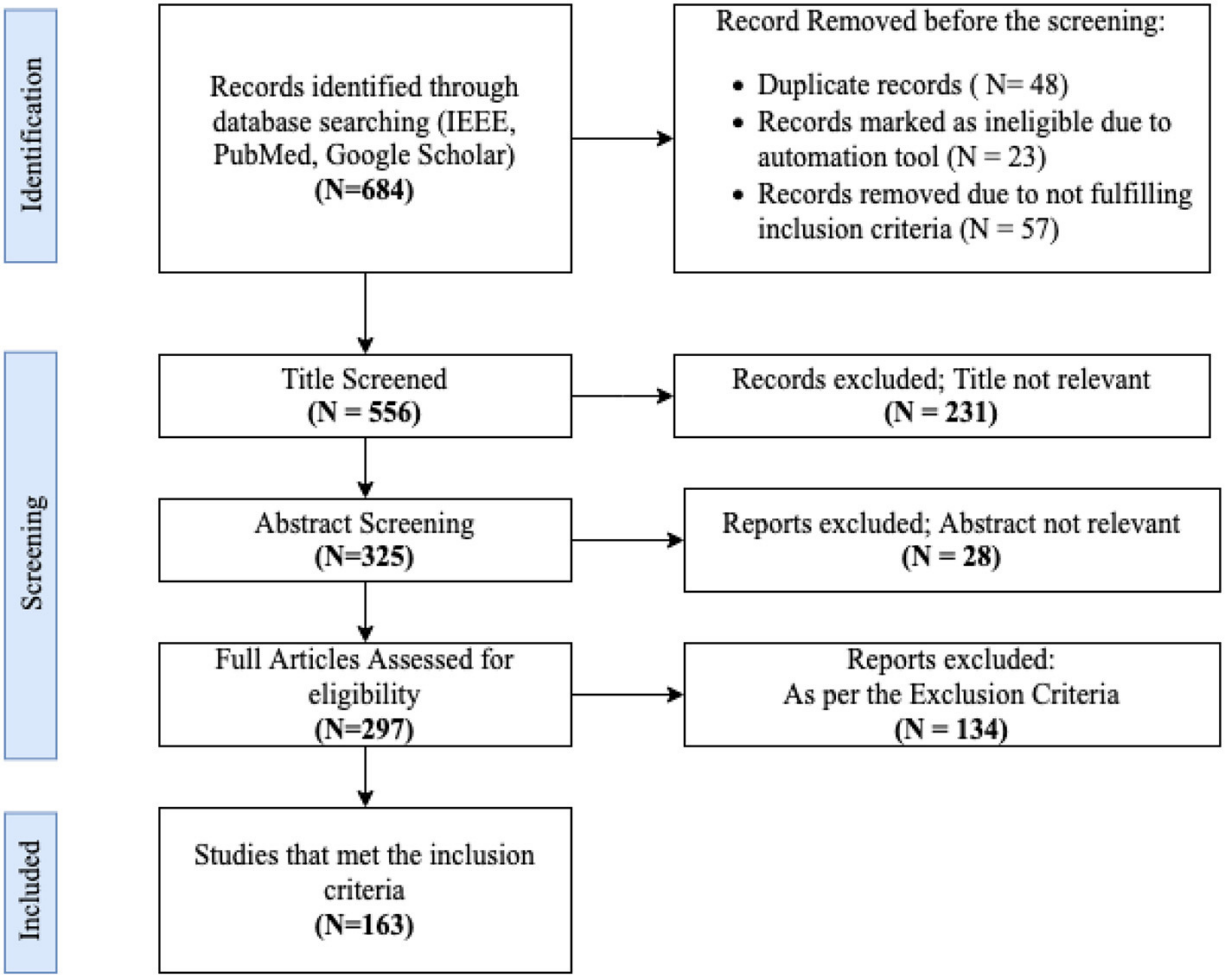
Flowchart illustrating the study selection process, with numbers denoting the count of studies at each phase.

Articles are initially screened based on their title and abstract. Those meeting the inclusion criteria have their full text retrieved for a more detailed evaluation. The criteria for inclusion encompass articles discussing machine learning techniques, especially FSL and its associated strategies, in the context of EEG signal analysis. Exclusions are made for articles not in English, those not peer-reviewed, or those not primarily focused on EEG signal analysis. The complete results are summarized in Figure 2.

The methodology adopted in this paper is outlined as follows:

- Adherence to the Preferred Reporting Items for Systematic Reviews and Meta-Analyzes (PRISMA) guidelines ensures the transparency and rigor of the search strategy, screening procedures, and data extraction (Liberati et al., 2009).
- Data extraction is executed based on predefined criteria, encompassing aspects like the publication year, study design, sample size, dataset employed, EEG feature extraction method, machine learning technique, and FSL strategy. The review assesses the maturity and applicability of each FSL strategy in EEG signal analysis, pinpointing existing challenges.
- A qualitative analysis of the extracted data offers a snapshot of the prevailing state-of-the-art in FSL and its strategies for EEG signal analysis. This review's contributions extend to discerning current trends, challenges, and potential solutions in the realm of FSL for EEG signal analysis.

The criteria guiding the inclusion and exclusion of articles in the selection process are summarized in Table 3.

**Table 3:** Inclusion and exclusion criteria.

**Inclusion criteria**	**Exclusion criteria**
Articles published within the past 10 years	Studies involving multimodal signals combined with EEG, such as Electrocorticography (ECoG), Functional Magnetic Resonance Imaging (fMRI), Electrocardiography (ECG), and Magnetoencephalography (MEG)
Studies focusing specifically on non-invasive EEG signals	Articles lacking robust metrics to validate the efficacy of the proposed method in classification tasks using either DA, TL, or SSL
Articles discussing the application and benefits of Data Augmentation (DA), Transfer Learning (TL), or Self-Supervised Learning (SSL) techniques	-
Studies employing DA, TL, or SSL techniques in EEG paradigms, including but not limited to emotion recognition, motor imagery classification, event-related potential classification, etc	-

## 3 Results

This section presents the results based on the above-discussed comprehensive methodology, encompassing various techniques, including Data Augmentation (DA), Transfer Learning (TL), and Self-Supervised Learning (SSL). Collectively, these techniques form a robust framework for training models to comprehend the nuances of out-of-distribution data, enabling domain adaptation and facilitating unsupervised representation learning. The amalgamation of these methodologies not only addresses the challenges of few-shot Learning but also underscores their synergistic potential in advancing the field of EEG signal processing. Together, they pave the way for a more profound understanding of cognitive neuroscience and related domains by harnessing the power of limited data resources and extending the boundaries of knowledge acquisition.

### 3.1 Data augmentation

Data Augmentation (DA) is a technique used to create more training examples by modifying existing data. This helps solve problems like having too few examples or having data that changes a lot. Having a robust model is challenging for the following reasons that DA aims to address while generating near real-world synthetic data (Simonyan and Zisserman, 2014; He et al., 2016).

1. The datasets available in EEG are smaller and imbalanced, and hence, samples of some controlled classes would be less; for example, studying epileptic seizure detection would not have an equal distribution of subjects with seizures as subjects without subjects such as used by Andrzejak et al. (2001).
2. It is hard to acquire diverse datasets representing a large proportion of real-world human demographics. This is due to the expensive setup to acquire quality EEG signals, which requires a controlled and stable environment for EEG signal capturing using highly sophisticated EEG sensors.
3. Due to limited and less diverse data, models may overfit and yield poor performance on unseen data, especially on new subjects or sessions.
4. The model trained can also be unstable, i.e., small fluctuations in the input can yield very different predictions.

**Figure 3:**
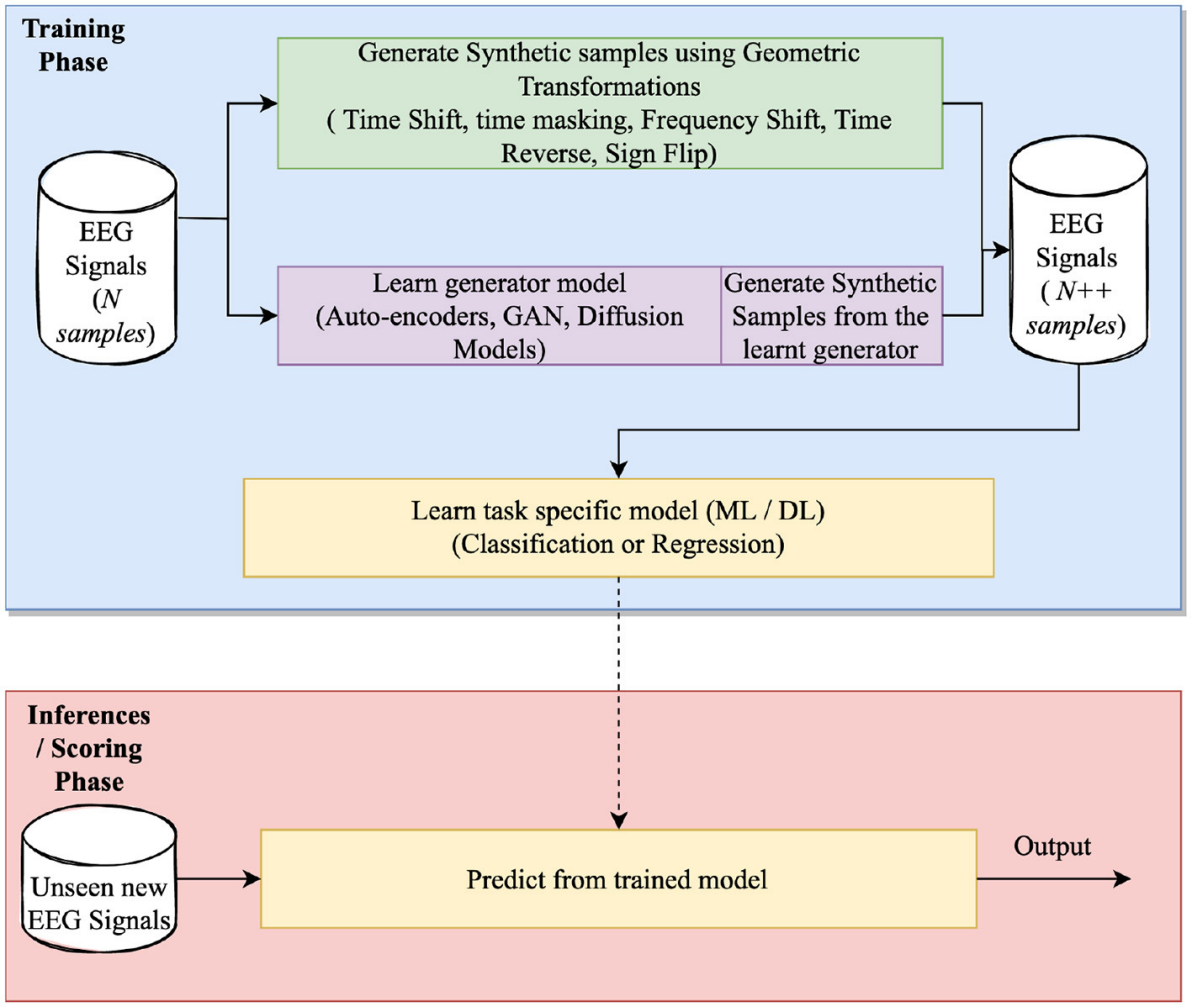
Schematic representation of an algorithm designed for generating synthetic EEG signals.

This section collectively analyzes the work done in DA for EEG signal processing while aggregating them by the techniques and the characteristics they aim to address. Figure 3 details a three-phase DA process designed to generate synthetic EEG signals with the goal of curating a diverse dataset that closely mirrors real EEG data. DA focuses on the training phase, which involves generating synthetic samples resembling the input samples by learning a generation function or manipulating existing samples.

#### 3.1.1 Characteristics of data augmentation

The robustness of DA can be explicated through its adept handling of diverse variances and the attributes inherent in the transformed samples. Researchers commonly focus on specific characteristics for transformation, aiming to maintain invariance to these features. In the pursuit of inferring the characteristics from the research, a meticulous set of steps was followed to discern the nuances of the methodologies employed. This process involves delineating results by subject, where the methodology is presumed to be exclusively tested for session invariance. Conversely, when results are aggregated across all subjects, it is reasonable to deduce that the methodology exhibits invariance to both session and subject. Additionally, if the methodology explicitly addresses how the class distribution is utilized for augmenting samples, whether through distribution balancing or separate augmentation per class, it implies class invariance. These characteristics are explicitly delineated in the research or inferred from the presentation of results and methodology, pertaining to Subject, Session, or Class Invariance.

1. *Subject invariance:* EEG signals often encapsulate features indicative of individual subjects. Consequently, a generator trained on such datasets becomes sensitive to these subject-specific features. An optimal generator, Subject Invariant Generator, should adeptly filter out these features. Both Aznan et al. (2020) and Panwar et al. (2020) proposed a Wasserstein Generative Adversarial Network (WGAN) for Rapid Serial Visual Presentation (RSVP). They used a gradient penalty to synthesize EEG data.
  Aznan et al. (2020) introduced the Subject Invariant Generative Adversarial Network (SIS-GAN) to generate synthetic EEG signals. The objective was to remove subject-specific elements while preserving Steady-State Visually Evoked Potential (SSVEP) frequencies. The architecture of SIS-GAN included a generator, discriminator, auxiliary classification network, and a pre-trained subject-biometric classifier. The synthetic data improved the performance of SSVEP frequency classification models.
  The study, conducted by Cimtay and Ekmekcioglu (2020), underscores the imperative of enhancing subject-independent recognition accuracy employing pre-trained Convolutional Neural Networks (CNNs). In lieu of relying on spectral band power characteristics, the authors employed windowed, adjusted, and normalized raw Electroencephalogram (EEG) data. The utilization of deep neural networks obviated the necessity for manual feature extraction, potentially revealing novel features. To mitigate false detections, a median filter was incorporated into the methodology. The proposed approach yielded mean cross-subject accuracies of 86.56 and 78.34% on the SEED dataset (Miller et al., 2014) for two and three emotion classes, respectively. Furthermore, it demonstrated a mean cross-subject accuracy of 72.81% on the DEAP dataset (Koelstra et al., 2011) and 81.8% on the LUMED dataset (Ekmekcioglu and Cimtay, 2021) for two emotion classes.
2. *Session invariance:* Achieving session invariance requires a generator capable of filtering out session-specific features from a signal. This generator's task is to retain only the essential information within the feature space while disregarding session-related variations.
  In the realm of person identification through EEG brain activity using deep learning, challenges were encountered by Özdenizci et al. (2019a). In response to these challenges, the authors proffered a solution employing invariant representation learning. Their approach encompassed the incorporation of an adversarial inference technique, aiming to foster the development of session-invariant and subject-invariant representations endowed with longitudinal applicability. In the context of within-session person identification models, the authors documented noteworthy accuracies of 98.7% ± 0.005, 99.3% ± 0.003, and 98.6% ± 0.006 for Sessions 1, 2, and 3, respectively. These outcomes were derived from the analysis of 2,760 half-second epochs, employing a 20% test split. The evaluation metrics employed facilitated a comprehensive assessment of model performance within individual sessions. Furthermore, in the domain of across-session person identification, when subjected to evaluation on an independent session, the model exhibited notable accuracies of up to 72% for 10-class person identification. This assessment was based on the analysis of 13,800 half-second epochs, with discernible enhancements of up to 6% attributed to adversarial learning and its influence on two sessions' invariance.
3. *Class invariance:* In addition to filtering out subject and session-specific characteristics, achieving class invariance in classification problems requires a generator to eliminate class-related features from the signal effectively.
  Rommel et al. (2021) introduced an advanced automated differentiable DA approach for EEG data, comparing class-wise augmentation to class-agnostic augmentation. Their methodology introduced novel EEG augmentations to aid model training in scenarios with limited labeled data.

#### 3.1.2 Data augmentation techniques

In this section, a comprehensive overview of various data augmentation techniques for EEG signals emerges from an extensive review of the selected papers. Data augmentation enhances the robustness and generalization of machine learning models by artificially expanding the training dataset. Based on the analysis, a proposed taxonomy outlines different data augmentation techniques tailored explicitly for EEG signal processing. These techniques encompass various approaches, including temporal augmentation, spatial augmentation, frequency-domain augmentation, and hybrid methods that combine multiple augmentation strategies. Each technique offers unique benefits and addresses specific challenges associated with EEG data, ultimately contributing to improved performance and adaptability of EEG-based machine learning models.

**Figure 4:**
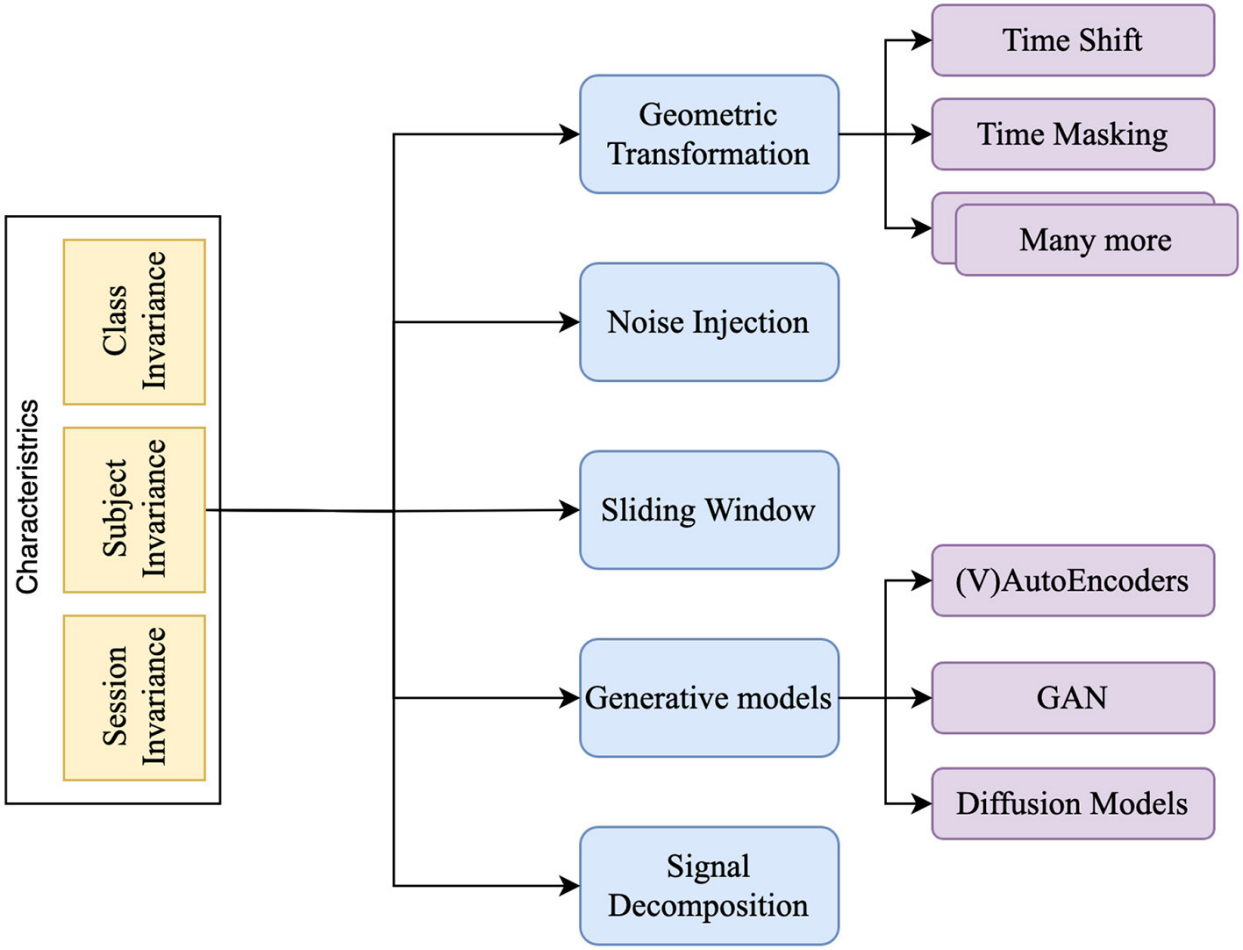
Taxonomy of Data Augmentation techniques for EEG signal processing.

Figure 4 shows the taxonomy of DA techniques explored in the literature. All the research in EEG focuses on improving the robustness of the model on unseen data while showcasing the improvement with and without augmentation. All techniques discussed further use this mechanism only to prove the effectiveness of the augmentation technique.

1. *Geometric Transformation (GT)* : Flipping, rotation, and cropping were common operations that altered the shapes of images, serving as visual representations of physical information encompassing both direction and contour. These techniques found wide application in speech signal processing (Cui et al., 2015) and computer vision (Paschali et al., 2019). In the context of EEG signals, the research by Krell and Kim (2017) highlighted that standard DA approaches, specifically in geometric transformations and noise injection (NI), did not adequately address variations in the signal-to-noise ratio (SNR) observed across multiple trials involving the same subject. The discussed techniques did not explicitly address whether they were designed to be session/subject invariant or class invariant.
  Table 4 outlines the typical transformations that are applied to raw time series data within the context of geometric transformations. Table 5 presents a comprehensive overview of prominent modeling techniques for EEG signal classification utilizing various geometric transformations, which have demonstrated the effectiveness of data augmentation in enhancing model generalization across sessions, subjects, and classes. While numerous studies have validated the efficacy of data augmentation and its necessity, none of these works have examined the statistical significance of the augmented signal. The techniques comparing pre- and post-augmentation (relative to the classification metric employed in the paper) demonstrate effectiveness for session and subject independence but do not assess whether the augmented session deviates from the original session or the same for the subject and class.
  Shovon et al. (2019) proposed a multi-input Convolutional Neural Network (CNN) for modeling Motor Imagery (MI) classification. The method converted each signal channel into an image representation using Short Time Fourier Transform (STFT). To address the data scarcity problem, rotation, flipping, and zooming were applied to the images. Experimental results on motor imagery datasets demonstrated accuracies of around 97 and 89.19% on the test split. However, the scalability of this technique might not have been optimal with higher dimensional EEG datasets. Freer and Yang (2020) introduced different augmentation methods for Motor Imagery (MI) classification tasks using a Convolutional Long-Short Term Memory (C-LSTM) network based on filter bank common spatial patterns (FBCSP). Training the model with data augmentations such as Noise Addition, Constant Multiplication, sign flip and frequency shift improved the model without augmentation by 5.3% on “BCIC IV—dataset—2a” (Brunner et al., 2008).
2. *Noise injection (NI)*: Gaussian white noise is added to the original signal or the generated features as a common DA technique. Since EEG signals exhibit a low signal-to-noise ratio, adding excessive noise can degrade the original signal. Therefore, applying NI requires careful consideration. Research works listed in Table 6 employ Noise Injection in training protocols and demonstrate performance improvements compared to models without noise injection. Despite the effectiveness of noise injection in data augmentation, a key open question remains: How much noise should be added, and how the efficacy of the augmented signal be intrinsically measured post adding noise?
3. *Sliding window (SW)* : Models typically utilize the complete signal for classification problems. When applied to small datasets, the sliding window mechanism creates multiple instances from a single sample by setting the *window size* to a value smaller than the original signal length. Table 7 lists studies that generate multiple instances from a single sample using SW for EEG signal classification and demonstrate performance improvements with the sliding window approach. An argument could be made that the sliding window may not be considered an effective enhancement technique because it doesn't significantly change the original signal. However, when looking at EEG data as a series of time-based signals, it's important to recognize that each segment over time may show some differences. Despite these variations, the key features used for classification remain somewhat similar across all time segments. Choosing the best window size becomes a subjective task depending on the dataset's characteristics, making it a hyperparameter that requires careful adjustment by researchers.
4. *Generative models*: Unlike deterministic transformation techniques, generative models learn the underlying data distribution and generate samples from this distribution, offering a more robust approach that facilitates automatic end-to-end modeling. Determining whether the generated samples remain subject or session-invariant poses a challenge. Nonetheless, Table 8 highlights the effectiveness of these models in EEG signal classification and their corresponding improvements.
  a. *Generative adversarial network (GAN)*: GANs generate artificial data through adversarial learning. In this process, two sub-networks, the Discriminator (D) and the Generator (G), strive to match the statistical distribution of the target data (Goodfellow et al., 2014). The Discriminator differentiates between genuine and artificial input samples, whereas the Generator produces realistic artificial sample distributions to deceive the Discriminator. The output from the Discriminator provides the likelihood of a sample being genuine. Probabilities near 0 or 1 denote distinct distributions, while values around 0.5 suggest challenges in discrimination. GANs effectively produce synthetic data resembling real distributions, as illustrated in Equation (1):
(1) $$\begin{array}{l} \begin{array}{c} \underset{G}{\min}\underset{D}{\max}V\left(D,G\right)={𝔼}_{x\sim p_{\text{data}}\left(x\right)}\left[\log D\left(x\right)\right]\\ +{𝔼}_{z\sim p_{z}\left(z\right)}\left[\log\left(1-D\left(G\left(z\right)\right)\right)\right] \end{array} \end{array}$$
    Below are prominent GAN architectures adapted for EEG-specific data generation:
    (1) *Deep convolutional generative adversarial network (DCGAN)*: DCGAN introduces a novel architecture by replacing pooling layers with fractional-strided convolutions in the generator and employing stride convolutions in the discriminator (Zhang et al., 2020b). This design, combined with adversarial training, ensures adherence to feature distribution principles. Studies have demonstrated that DCGAN produces samples that are both diverse and closely resemble real data. For instance, in epilepsy seizure detection using EEG signals from the CHB-MIT dataset (Goldberger et al., 2000), a DCGAN-based augmentation was employed, followed by classification using ResNet50. This approach achieved a 3% performance improvement over non-augmented datasets (He et al., 2016).
    (2) *Wasserstein generative adversarial network (WGAN)*: WGAN addressed the challenge of discontinuous GAN divergence for generator parameters, which could result in training instability or convergence issues (Arjovsky et al., 2017). The key innovation was the adoption of the Earth-Mover distance (Wasserstein-l), as described in Equation (2):
(2) $$\begin{array}{l} W\left({\mathbb{P}}_{r},\;{\mathbb{P}}_{g}\right)=\underset{\gamma \in \Pi \left({\mathbb{P}}_{r}{\mathbb{P}}_{g}\right)}{inf},{\text{E}}_{\left(x,y\right)\sim \gamma }\left[∥x-y∥\right] \end{array}$$
      Here, π*P*_*r*_, *P*_*g*_ denoted the set of all joint distributions of (x,y) where *P*_*r*_ and *P*_*g*_ were the marginals. The joint distribution (x,y) signified the “mass” transferred from x to y to transition from the distribution *P*_*r*_ to *P*_*g*_. The WGAN value function, based on the Kantorovich-Rubinstein duality (Villani, 2008), was given by Equation (3):
(3) $$\begin{array}{l} \min G\max D\in D\underset{x\sim \mathbb{P}r}{𝔼}\left[D\left(x\right)\right]-\underset{\tilde{x}\sim \mathbb{P}g}{𝔼}\left[D\left(\tilde{x}\right)\right] \end{array}$$
    (3) *Conditional Wasserstein GAN (cWGAN)*: In emotion recognition tasks using the SEED (Miller et al., 2014) and DEAP (Koelstra et al., 2011) datasets, combining cWGAN with manifold sampling has enhanced classifier performance by ~10% (Luo et al., 2020). cWGAN, a variant of WGAN, incorporated the auxiliary label by introducing the real label *Y*_*r*_ to both the discriminator and generator. The generator combines the latent input *X*_*z*_ with *Y*_*r*_, while the discriminator forms a hidden representation by merging the real input *X*_*r*_ and generated input *X*_*g*_ with *Y*_*r*_. Equation (4) presents the objective function for cWGAN, that inferently learns latent representation *X*_*z*_ of the input *X*_*r*_.
(4) $$\begin{array}{l} \begin{array}{l} \underset{\theta_{G}}{\min}\underset{\theta_{D}}{\max}L\left(X_{r},X_{g},Y_{r}\right)=\\ \;{𝔼}_{x_{r}\sim X_{r},y_{r}\sim Y_{r}}\left[D\left(x_{r}|y_{r}\right)\right]-{𝔼}_{x_{g}\sim X_{g},y_{r}\sim Y_{r}}\left[D\left(x_{g}|y_{r}\right)\right]\\ -\lambda {𝔼}_{\hat{x}\sim \hat{X},y_{r}\sim Y_{r}}\left[{\left(||\nabla_{\hat{x}|y_{r}}D\left(\hat{x}|y_{r}\right)|{|}_{2}-1\right)}^{2}\right] \end{array} \end{array}$$
  b. *Autoencoders (AE)*: Autoencoder Rumelhart et al. (1985) referred to feed-forward neural networks that encoded raw data into low-dimensional vector representations through the encoder and then reconstructed these vectors back into artificial data using the other half of the network. Instead of outputting vectors in the latent space, the encoder of Variational Autoencoders (VAE; Kingma and Welling, 2013) produced parameters of a pre-defined distribution in the latent space for each input. The VAE then enforced constraints on this latent distribution, ensuring its normality. Compared with AE, VAE ensured that generated data adhered to a specific probability distribution by introducing structural constraints (Luo et al., 2020). Komolovaitė et al. (2022) introduced a synthetic data generator using VAE for Stimuli Classification employing EEG signals from healthy individuals and those with Alzheimer's disease. This technique exhibited a 2% improvement over models without non-augmented data.
  c. *Diffusion models*: VAE constrained a specific probability distribution in the latent space. Normalizing Flows learned a tractable distribution in which both sampling and density evaluation could be efficient and precise. However, Normalizing Flows were trained in a denoizing manner to capture an underlying distribution. Ho et al. (2020) outperformed GAN while devising a denoizing model and normalizing flows for image generation. Similarly, Hajij et al. (2022) employed a diffusion model-based generator for chest X-ray images to create synthetic data.
  The initial work, as illustrated above, utilized various generative models. Recent research has also shifted focus toward determining optimal tasks for training generative models, rather than exclusively focusing on signal reconstruction. It has led to developing models such as GANSER (Generative Adversarial Network-based Self-supervised Data Augmentation; Zhang et al., 2023). Unlike previous approaches that reconstructs the complete signal, GANSER emphasizes masking the data and predicting the masked part of the signal for pre-training. Subsequently, the classifier is fine-tuned over the pre-trained model to predict augmentation. In this process, 80% of the data is utilized for pre-training without labels, followed by fine tuning with all the labels. While the paper does not explicitly demonstrate improvement with and without the proposed method, it compares favorably with other state-of-the-art emotion recognition models and other generative method for DEAP Koelstra et al. (2011) and DREAMER Katsigiannis and Ramzan (2018), showcasing an improvement of ~4% across all classes compared to the respective state-of-the-art models.
5. *Signal decomposition*: Unlike other techniques discussed so far, DA in feature space is another technique that has shown some promising results. Generally, these techniques decompose a signal using methods such as Empirical Model Composition (EMD) or Fourier Transform (FT), transform the signal in the decomposed space, and then reconstruct the time domain signal. All the studies utilizing Signal Decomposition have augmented signals on a per-class basis, thereby establishing class invariance.
  Kalaganis et al. (2020) proposed a DA method based on Graph-Empirical Mode Decomposition (Graph-EMD) to generate EEG data, which combined the advantages of the multiplex network model and the graph variant of classical empirical mode decomposition. They used a graph CNN to implement the automated identification of human states while designing a continuous attention-driving activity in a virtual reality environment. The experimental results demonstrated that investigating the EEG signal's graph structure could reflect the signal's spatial characteristics and that merging graph CNN with DA produced more reliable performance. Zhang et al. (2019) suggested a new way to classify EEG data by combining DL and DA. The classifier consisted of Morlet wavelet features as input and a two-layered CNN followed by a pooling layer NN architecture. The author used EMD on the EEG record, mixing their Intrinsic Mode Functions (IMF) to create a new synthetic EEG record.
  Huang et al. (2020) proposed three different augmentation techniques: Segmentation, Time domain exchange, and Frequency domain exchange to generate more training samples. Combined with a CNN for training the classification model, these techniques yielded a gain of 5–10 % accuracy compared to pre-augmentation. Schwabedal et al. (2018) proposed a novel augmentation technique called FT Surrogates. FT Surrogates generated new samples by changing the signal phase by decomposing the signal using the Fourier Transform (FT) and then reconstructing it into the time domain using the Inverse Fourier Transform (IFT). The premise that stationary linear random process sequences are uniquely described by their Fourier amplitudes, while their Fourier phases are random values in the interval [0, 2π), drove this approach.

**Table 4:** Common Geometric Augmentations for EEG data.

**Augmentation**	**Space**	**References**	**Description**
FTSurrogate	Frequency	(Schwabedal et al., 2019)	Randomize Fourier phases of all channels.
FrequencyShift	Frequency	(Rommel et al., 2021)	Randomly shift PSD of all channels.
SignFlip	Time	(Rommel et al., 2021)	Randomly flip the sign of all channels.
TimeReverse	Time	(Rommel et al., 2021)	Randomly reverse the axis of time in all channels.
ChannelsSymmetry	Spatial	(Deiss et al., 2018)	Randomly swap signals between hemispheres.
ChannelsDropout	Spatial	(Saeed et al., 2021)	Randomly set signals of channels to zero.
ChannelsShuffle	Spatial	(Saeed et al., 2021)	Randomly permute signals of channels.
SensorsRotation	Spatial	(Krell and Kim, 2017)	Interpolate signals on rotated positions.
TimeShift	Time	(Mohsenvand M. N. et al., 2020; Mokatren L. S. et al., 2020)	Translate the entire signal by –0.5 to 0.5.
TimeMasking	Time	(Cheng et al., 2020)	Replace a random section with zeros.

**Table 5:** DA using geometric transformation for EEG signal classification. MI, Motory Image; ER, Emotion Recognition; SS, Sleep Staging.

**References**	**EEG paradigm**	**Dataset**	**DA characteristic**	**Feature space**	**Model**	**Improvements post augmentation**
Shovon et al. (2019)	MI	BCIC II (III); BCIC IV (2b)	Subject Variance	Time Frequency	CNN	NA to 89.9%; NA to 97 %
Freer and Yang (2020)	MI	BCIC IV (2a)	Session Invariance	Time Series	C-LSTM	Avg of ~14%
Mokatren L. S. et al. (2020)	ER	DEAP	Session Invariance	Wavelet	InceptionNet	Avg of 2.2 ~5%
Rommel et al. (2021)	MI, SS	Physionet SleepEDF	Class Invariance	Time Frequency	Auto Augment	NA

**Table 6:** DA using noise injection (NI) for EEG signal classification. MI, Motory Image; R, Emotion Recognition; MWD, Mental Workload Detection.

**References**	**EEG paradigm**	**Dataset**	**DA char**	**Feat space**	**Model**	**Improvement**
Wang et al. (2018)	ER	SEED: MANHOB- HCI	Session Invariant	DE	SVM	40.8 to 45.4%
Salama et al. (2018)	ER	DEAP	-	Time Series	3D-CNN	79.11 to 89.4%
Li et al. (2019c)	MI	BCIC IV (2a): HGD	-	Spectral Image	CP-MixedNet	Avg of 1.1%
Yin and Zhang (2017)	MWD	Proprietary	Session Invariant	SAE	RNN	34.2 to 75%
Kuanar et al. (2018)	MWD	Proprietary	-	Spectral Image	CNN	NA to 93%
Zhang et al. (2021a)	MI	BCIC IV (2a & 2b);	Subject Invariant	Time Series	Inception Net	90 to 95%

**Table 7:** DA using sliding window for EEG signal classification.

**References**	**EEG paradigm**	**Dataset**	**DA char**	**Feat space**	**Model**	**Improvement**
Majidov and Whangbo (2019)	MI	BCIC IV (2a & 2b)	NA	Time Series	RM Classifier	NA to 80.4~82.39%
O'Shea et al. (2017)	SD	Clinic Dataset	NA	Spatial Temporal	CNN	NA to 97%
Mousavi et al. (2019)	SS	SleepEDF	Subject and Session Invariant	Time Series	CNN	82.9 to 85%
Avcu et al. (2019)	SD	Clinic Dataset	NA	Time Series	CNN	NA to 93%
Tayeb et al. (2019)	MI	BCIC IV (2b)	NA	Spectral Image	CNN	NA to 84%
Tsiouris et al. (2018)	SD	CHB-MIT	Subject Invariant	Spatial Temporal	LSTM	70 to 80 %

MI, Motory Image; SD, Seizure Detection; SS, Sleep Staging; Feat Space, Feature Space; DA Char, DA Characteristics.

**Table 8:** DA using generative models for EEG signal classification.

**References**	**EEG paradigm**	**Dataset**	**DA characteristic**	**DA technique**	**Feat space**	**Model**	**Improvements**
Wei et al. (2019)	SD	CHB-MI	NA	WGAN	Time Series	GCNN	72.11 to 95.89 %
Chang and Jun (2019)	ER	Proprietary	Subject and Session Invariant	GAN	Time Series	DNN	NA to 98.4%
Luo et al. (2020)	ER	SEED; DEAP	Subject Invariant	cWGAN + sVAE	PSD + DE	SVM	NA to 90.8 %
Zhu et al. (2018)	ER	JAFFE	Subject and Session Invariant	Cycle GAN	Time Series	CNN	Avg 3.7 ~8%
Schlögl (2003)	MI	BCIC II (3)	NA	cDCGAN	Time Freq	CNN	78 - 83 %
Zhang et al. (2020b)	MI	BCIC IV (1, 2b)	NA	DCGAN	Spectral Image	CNN	74.5 to 83.2 % 80.6 to 93.2 %
Fahimi et al. (2020)	MI	Prop- rietary		cDCGAN	Spectral Image	Avg 3.22 ~5.45%	EEGNet
Lawhern et al. (2018)	RSVP	BCIT X2 RSVEP	NA	WGAN	Time Series	CNN	NA
Piplani et al. (2018)	MWD	Prop- rietary	NA	GAN	Spectral Image	Boosting	90 to 95 %
Hartmann et al. (2018)	MI	NA	NA	GAN	Spectral Image	k-NN	NA
Luo and Lu (2018)	ER	SEED	Subject Invariant	GAN	Spectral Image	CWGAN	Avg 3~20 %
Zhang et al. (2018)	MI	BCIC IV (2b)		GAN	Spectral Image	CNN	77 to 79 %
Chang and Jun (2019)	ER	CHB MIT	Subject and Session Invariant	GAN	Time Series	GAN	97 to 98 %
Zhang et al. (2019)	MI	BCIC IV (2b)	Subject Invariant	GAN	Time Series	CNN + LSTM	NA to 76%
Panwar et al. (2019)	RSVP	BCIT X2 RSVEP	NA	GAN	Time Series	CNN	0.7 ~2%
Arỳ et al. (2022)	ER	MAH- NOB HCI	NA	AE	Wavelet	CNN	Avg ~22 %
Aznan et al. (2019)	SSVEP	Prop- rietary	Subject Invariant	VAE	Time Series	CNN	Avg ~35%
Zhang et al. (2023)	ER	DEAP, DREAMER	Subject, session, Class Invariant	GAN	Time Series	CNN	NA

SD, Seizure Detection; ER, Emotion Recognition; MI, Motory Image; SSC, Sleep Staging Classification; MWD, Mental Workload Detection; RSVP, Rapid Serial Visual Presentation; SSVEP, Steady-State Visually Evoked Potentials; NA, not explicitly provided in the paper or can't be inferred.

### 3.2 Transfer Learning for EEG signal classification

Transfer Learning (TL) is a method where a model trained on a lot of data in one area (source) is used to help learn from less data in another area (target). This is useful for understanding brain signals.

1. Acknowledging the inherent dynamism in human physiology and psychology is crucial, as individuals undergo constant physical and mental fluctuations.
2. The profound inter-individual variability in human behavior and characteristics accentuates the need for continuous model updates.
3. The acquisition of EEG signals, essential for decoding brain activity, introduces further complexity due to sensor variations from diverse manufacturers.

**Figure 5:**
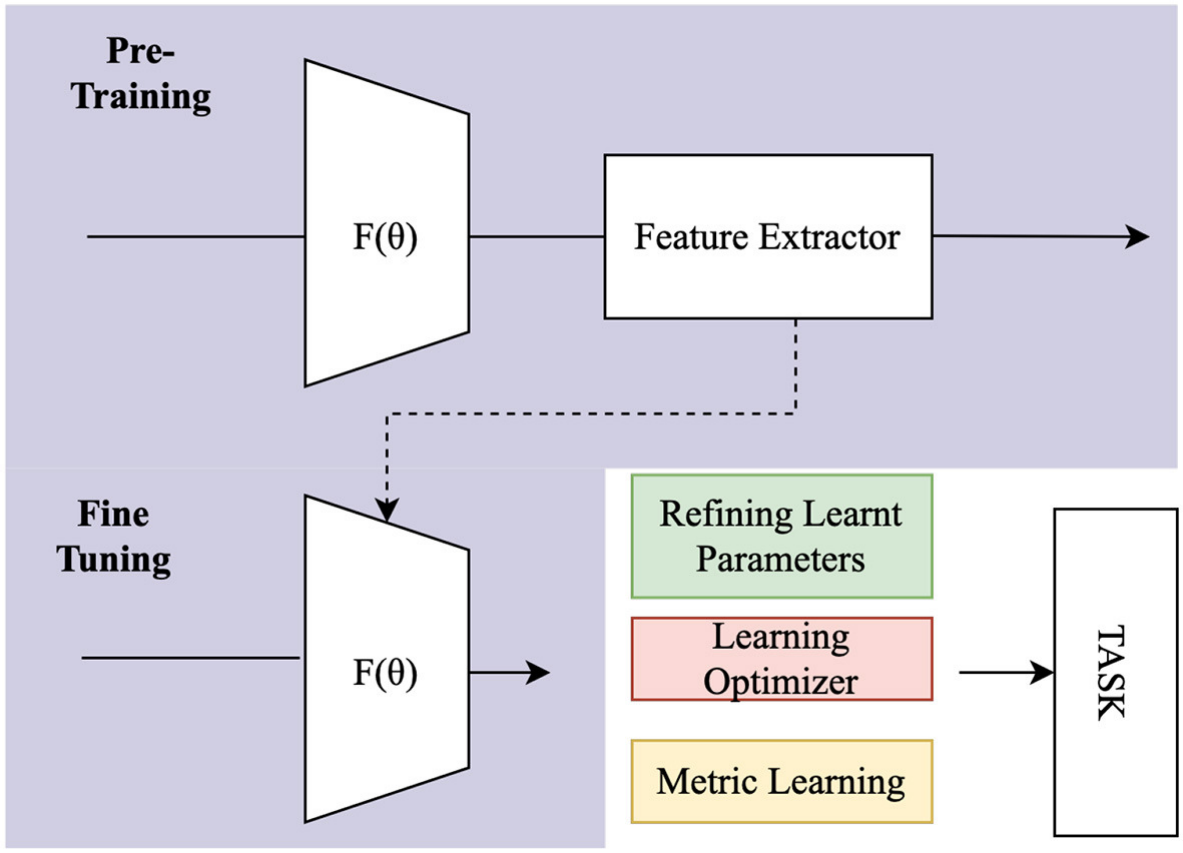
Process flow of Transfer Learning.

Hence, the imperative arises to prioritize ongoing data transfer, steering away from retraining models from scratch. This multifaceted consideration underscores the importance of embracing Transfer Learning not only as a methodological necessity but as a dynamic and adaptive tool for the nuanced exploration of human brain signals in the ever-evolving landscape of neuroscience. The source and target tasks are the same in domain transfer learning, but the source and target domains are distinct. For example, they can differ due to EEG sensors, different subjects, or even different but similar tasks. Transfer Learning is common in FSL, where prior information is transferred from the source task to the few-shot task through transfer learning methods (Luo et al., 2017; Azadi et al., 2018; Liu et al., 2018). Figure 5 illustrates the entire transfer learning procedure, involving pre-training using comparable datasets and fine tuning the pre-trained model for the intended job.

#### 3.2.1 Transfer of characteristics

Transfer Learning transfers many data characteristics across the datasets. This section sheds light on the different transfer of characteristics, specifically in EEG signals, and the work done to them.

1. *Cross subject and session:* This approach leverages information from both other subjects (referred to as “source domains”) and previous sessions to help calibrate a new subject and session (referred to as the “target domain”). Cross-subject knowledge can be transferred by incorporating data from multiple subjects who perform the same task using the same EEG equipment to enhance learning. Similarly, preserving consistency between sessions, where data from earlier sessions are utilized to calibrate the data from the present session, aids in capturing temporal relationships and enhances generalization. This combined approach enables the model to benefit from a broader range of data, reducing the dependency on subject-specific or session-specific labeled examples and enhancing the overall few-shot learning performance.
  Yang et al. (2019) examined cross-subject classification of emotions on DEAP (Koelstra et al., 2011) and SEED (Miller et al., 2014). Ten linear and non-linear features were extracted from each channel and assembled. These included the standard deviation, PSD (alpha, beta, gamma, and theta), sample entropy, and wavelet entropy. The Hjorth coefficients (activity, mobility, and complexity) were also considered. Then, significance tests and sequential backward feature selection chose the features and trained a classifier using SVM with an RBF kernel. Fahimi et al. (2019) trained a CNN model using EEG signals of the source subjects, followed by fine tuning using the calibration data. The model inputs EEG frequency bands, specifically delta, theta, alpha, beta, and gamma bands, subsequent to filtering through a Bandpass filter in this methodology. The suggested technique exhibits potential for generalization across diverse subjects. Li et al. (2019a) proposed a neural network model for cross-subject/session EEG emotion recognition that did not require label information in the target domain. By reducing the classification error in the source domain and aligning the latent representations of the source and target domains, the neural network was improved as much as possible. It adjusted to the joint distribution in this manner. The first few layers altered the marginal distributions using adversarial training, and the last few layers altered the conditional distributions using association reinforcement.
  Song et al. (2018) suggested a Dynamical Graph Convolutional Neural Network (DGCNN) for classifying emotions dependent on the subject and those that were not. Differential entropy features from five distinct frequency bands were fed into the DGCNN. A node in the network represented each EEG channel. After graph filtering, a 1x1 convolutional layer learned what differentiated the five frequency bands. A ReLU activation function ensured that the outputs of the graph filtering layer were always positive. The study evaluated the proposed method using the DREAMER (Katsigiannis and Ramzan, 2018) dataset, resulting in recognition accuracies of 86.23, 84.54, and 85.02% for valence, arousal, and dominance prediction, respectively.
  Ning et al. (2021) introduced the SDA-FSL approach for cross-subject EEG emotion recognition. This method effectively addressed challenges posed by individual differences and limited information in EEG analysis. SDA-FSL integrated key components, including a CBAM-based feature mapping module, a domain adaptation module, and Prototypical Networks with an instance-attention mechanism. Evaluations on DEAP (Koelstra et al., 2011) and SEED (Miller et al., 2014) datasets demonstrated its superior performance in cross-dataset experiments. However, limitations such as data requirements and interpretability issues were noted.
2. *Cross device:* The data from the source EEG device (referred to as the source domain) is utilized to calibrate another EEG device (referred to as the target domain) in a manner where both EEG devices are standardized to perform the same task using common electrodes.
  Lan et al. (2018) utilized various EEG devices, each with varying numbers of electrodes, to record data from DEAP (Koelstra et al., 2011) and SEED (Miller et al., 2014), datasets that contained different numbers of individuals. The study encompassed the 32 channels shared by the two datasets, each comprising merely three trials—positive, neutral, and negative outcomes, respectively. The final feature set for each channel required the extraction and combination of five distinct frequency bands. The study demonstrated that applying domain adaptation (Yan et al., 2017), particularly techniques such as transfer component analysis (Pan et al., 2010) and maximal independence domain adaptation, significantly augmented classification accuracy. DL approaches were shown to enhance cross-device TL in BCIs greatly. EEG data were frequently transformed into images before inputting into the models, rendering EEG signal outputs consistent across different devices.
  In a similar vein, Siddharth et al. (2019) engaged in cross-dataset emotion categorization, employing various modalities such as EEG, Electrocardiography (ECG), and Facial Expression Recognition (FAR). The paper briefly discussed their EEG-based deep learning technique for emotion classification, training on DEAP (Koelstra et al., 2011) and testing on the MAHNOB-HCI dataset (Soleymani et al., 2011). This approach remained effective across datasets with diverse numbers and locations of electrodes, as well as variable sampling rates. Power spectral densities (PSDs) of theta, alpha, and beta bands from EEG signals were extracted for all trials—the suggested technique generated topographic PSD images for each trial, consolidating information from multiple EEG devices. The alpha blending ratio was used to weigh each topography as a component of a color image. VGG-16 (Tammina, 2019) was employed to extract 4,096 features from the images, which were subsequently reduced to 30 through Principal Component Analysis (PCA). After pre-training, the author applied an extreme learning machine as the final classification classifier.
  An advanced CNN model, Inception-ResNet-v2 (Szegedy et al., 2016), was utilized by Cimtay and Ekmekcioglu (2020) to transfer information across subjects and datasets. Adding Gaussian random noise expanded the number of channels from 75 to 80 in Inception-ResNet v2's input data size, which is (N1, N, 3), where N1 is the number of EEG channels and N *geq* 75 is the number of time domain samples. An 80x300x3 matrix was created for each trial, which was then sent into Inception-ResNet-v2 for further processing. After Inception-ResNet-v2, the work included a global average pooling layer and five dense layers for classification purposes.
3. *Cross task:* Calibration for a new task is made more accessible by using labeled data from prior activities that are comparable or relevant (source domains; the target domain). Left-and Right-handed MI data calibrates the foot and tongue Motory Image (MI). In most tasks, the subject and the EEG instrument remain the same.
  He and Wu (2020) compared Label Alignment (LA) with Reiman Alignment (RA) and Euclidean Alignment (EA, Eisenhart, 2016). RA and LA assumed that the source and target domains shared identical features and label spaces in cross-task transfers. However, this assumption held only in certain practical implementations. LA accommodated source domains that had distinct label spaces from the target domains. For instance, during the calibration of a target subject for Motor Imagery-based Brain-Computer Interfaces (MI-based BCIs), source subjects might have performed tasks such as left and right-hand MIs, while target subjects might have engaged in foot and tongue MIs. LA identified the EEG channels from the source that most closely resembled the EEG channels of interest in the target. This method estimated covariance matrices for each target class. It then recentered each source domain's classes to their expected mean. The alignment of these trials facilitated feature extraction and classification in both Euclidean and Riemannian spaces. Since LA needed only one labeled sample from each class in the target domain, researchers could use it as a preprocessing step before implementing other feature extraction and classification techniques.
  Zheng et al. (2020) made significant contributions to the field of motor-imagery brain-computer interface systems (MI-BCI) by addressing key challenges related to task transfer and enhancing the usability of MI-BCI applications. They introduced a novel approach that enlarged the command set by incorporating combinations of traditional MI commands, thereby expanding the potential applications of MI-BCI. Moreover, they developed a transfer learning-based algorithm for feature extraction that demonstrated remarkable results. This algorithm effectively reduced the calibration time required for data collection and model training while improving classification accuracy, particularly for low-quality datasets. Notably, the authors highlighted the practical implications of their work by showing how it made MI-BCI more user-friendly for subjects, eliminating the need for extensive training to adapt to MI tasks. Additionally, the findings suggested that this algorithm outperformed traditional methods, especially in scenarios with limited training samples and suboptimal performance in conventional algorithms. However, they did not explore looking beyond MI in this work.

#### 3.2.2 Techniques for Transfer Learning

**Figure 6:**
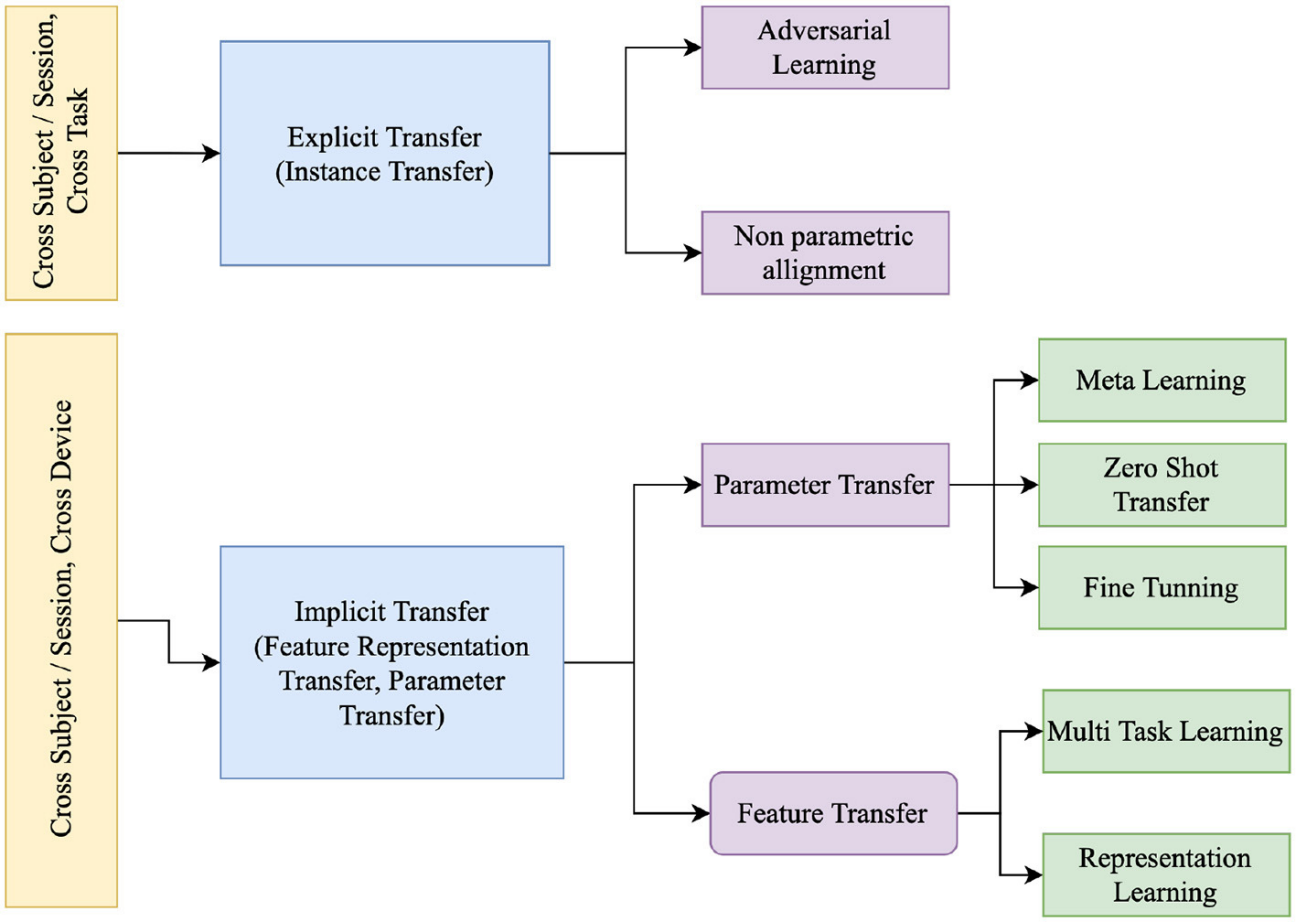
Taxonomy of Transfer Learning (Zhang et al., 2020c; Ko et al., 2021).

The categorization of Transfer Learning (TL) by Ko et al. (2021) comprises two principal classifications: *Implicit Transfer Learning (ITL)* and *Explicit Transfer Learning (ETL)*. ETL explicitly addresses distinctions between domains, such as subjects or training sessions, with the objective of mitigating disparities through the alignment of feature spaces during the training process. On a more detailed level, ITL encompasses methodologies such as Representation Learning (Schirrmeister et al., 2017), Fine Tuning (Andreotti et al., 2018; Fahimi et al., 2019; Zhao et al., 2019; Raghu et al., 2020), and Meta-Learning (Finn et al., 2017), as illustrated in Figure 6. Similarly, ETL involves techniques like Non-Parametric Alignment (Gretton et al., 2012), or Adversarial Learning (He and Wu, 2019). Zhang et al. (2020c) further categorizes TL into instance transfer, parameter transfer, and feature transfer. It's important to note that domain adaptation through feature and parameter transfer, occurring without explicit alignment, falls under ITL. Similarly, instance transfer, involving explicit alignment of instances to the source domain, is equivalent to ETL.

1. *Explicit learning*
  (a) *Non-parametric alignment*: In the context of EEG signal processing, addressing distributional disparities across subjects or sessions is paramount. Non-parametric alignment emerges as a pivotal technique in this regard. Unlike its parametric counterparts, non-parametric alignment does not adhere to predetermined models or specific distributional assumptions. Instead, it directly aligns the distributions of EEG signals from different domains, leveraging the inherent characteristics of the data. This flexibility enables it to accommodate diverse and intricate EEG data structures, enhancing its applicability in EEG-based transfer learning scenarios. By bridging the distributional gap between source and target domains, non-parametric alignment ensures seamless and effective knowledge transfer, even amidst varying data distributions. A comprehensive overview of research employing non-parametric alignment for EEG signal processing can be found in Table 9, underscoring the technique's versatility and efficacy.
  (b) *Adversarial Training*: Adversarial Training employs a distinctive strategy where a discriminator network distinguishes between samples from the source and target domains. Simultaneously, the feature extractor network is designed to produce domain-invariant representations. This dual mechanism ensures cohesive alignment between the source and target domains. The objective is 2-fold: to diminish the discriminator's capacity to differentiate domains and to ensure that the feature extractor captures representations shared by both domains. Table 9 presents a detailed overview of how Adversarial Training is applied in Explicit Domain Adaptation, highlighting its role within the Explicit Learning (EL) Type framework.
2. *Implicit Learning*
  (a) *Parameter transfer*: Implicit learning constitutes a foundational facet of transfer learning, encompassing pre-learned parameters, latent spaces, or task-agnostic representations. This technique bolsters performance and facilitates efficient adaptation across diverse machine learning applications, particularly within EEG signal processing. Amid various transfer learning strategies, fine tuning emerges as a potent approach to tailor a pre-trained model from a source domain to a target domain burdened by scant labeled data. Refining the model's parameters through a smaller labeled dataset or a few-shot learning arrangement markedly enhances its capability to unearth implicit insights from EEG signals. In contrast, the paradigm of zero shot transfer introduces an innovative avenue for prognosticating implicit information within EEG signals, obviating the necessity for explicit training on the specific information. This methodology capitalizes on semantic representations or attributes tied to the intended implicit information, enabling the model to extrapolate knowledge from kindred domains or explicit learning tasks. Furthermore, the utility of meta-learning becomes evident in swift adaptation to nascent implicit learning undertakings, even in the presence of limited labeled instances. Through meta-learner training on task distributions, each comprising only a handful of labeled samples, the model imbibes task-agnostic attributes, enabling agile assimilation of novel tasks within EEG-centric implicit learning.
    (1) *Fine tuning*: In this form of transfer learning, the model is first pre-trained on the source dataset and further fine-tuned on the (smaller) target dataset to learn its specific characteristics (Pan and Yang, 2009). There are various fine-tuning mechanisms that either fine tune the complete network end to end or fine tune either the last few layers (classification or regression heads) or specific layers of the network which are highlighted in Table 10.
    (2) *Meta transfer*: Meta-learning is like teaching a model how to learn quickly from new tasks or situations. It's especially helpful when there are only a few examples to learn from. The utilization of meta-learning has gained importance in machine learning domains and has recently extended its application to Brain-Computer Interfaces (BCIs) based on Deep Learning (DL; An et al., 2020a; Duan et al., 2020; Li et al., 2021a; Pati et al., 2023; Ng and Guan, 2024) as highlighted in Table 10.
      Duan et al. (2020) employed a Model-Agnostic Meta-Learning (MAML) approach (Finn et al., 2017), seeking optimal parameters adaptable to target data through gradient-based optimization across multiple subjects. The methodology involved parameter updates based on gradients during two distinct phases: meta-training and meta-test. Subsequently, fine-tuning was performed with a limited amount of target data. Notably, the susceptibility of MAML to overfitting prompted (Duan et al., 2020). To design shallow convolutional layers for feature extraction. This design choice, however, may limit the capacity of their method to sufficiently capture class-discriminative information.
      Recent advancements include the work of Pati et al. (2023), which demonstrated the effectiveness of optimization-based meta-learning for subject adaptation in low-data environments. They utilized an optimization-based meta-learning approach to tackle the problem of subject variability in EEG-based motor imagery classification. This method involves pre-training a model using data from multiple subjects and fine-tuning it on new subjects with limited data. The model employed is EEGNet, a compact convolutional neural network optimized for EEG signal classification. During meta-training, the model learns an initialization that can be quickly adapted to new tasks using a few gradient steps. This allows the model to generalize better to unseen subjects, significantly improving classification accuracy in low-data scenarios. The results showed that this approach outperformed traditional transfer learning methods, demonstrating the potential of meta-learning for enhancing BCI systems.
      In another notable work, Li et al. (2021a) applied Model-Agnostic Meta-Learning (MAML) to EEG motor imagery decoding, enabling rapid generalization to new users and sessions through few-shot learning and gradient-based optimization. Their approach significantly improves classification accuracy, demonstrating the potential of meta-learning in BCI applications. This approach enables rapid generalization to new users and sessions through few-shot learning and gradient-based optimization. MAML operates in two phases: meta-training and meta-testing. During meta-training, the model parameters are optimized such that a few gradient updates can lead to good performance on new tasks. This involves repeatedly training on small batches of tasks and then fine-tuning on new, unseen tasks with limited data. The results showed significant improvements in classification accuracy, indicating that MAML can effectively handle the variability in EEG signals across different sessions and subjects.
      Moreover, Han et al. (2024) proposed META-EEG, an advanced implicit transfer learning framework designed to tackle inter-subject variability in MI-BCIs. By incorporating gradient-based meta-learning with an intermittent freezing strategy, META-EEG ensures efficient feature representation learning, providing a robust zero-calibration solution. Comparative analysis reveals that META-EEG significantly outperforms baseline and competing methods on multiple public datasets, demonstrating robust performance and generalizability even with unseen subjects through a leave-one-subject-out cross-validation (LOOCV) training strategy. This state-of-the-art framework highlights the efficacy of meta-learning in achieving calibration-free MI-EEG classification.
      Despite these advancements, challenges remain, such as the risk of overfitting, the need for computational resources, and the difficulty in capturing complex class-discriminative features. Addressing these challenges requires further research into more robust and efficient meta-learning frameworks that can generalize well across different subjects and tasks without extensive computational overhead. Wu and Chan (2022) suggests that recent advancements in meta-learning, such as Reptile algorithms (Nichol et al., 2018), may not significantly enhance performance in EEG tasks, particularly motor imagery classification. Reptile is a first-order meta-learning algorithm designed for rapid adaptation to new tasks. It works by performing gradient descent steps on randomly sampled tasks to find a good initialization, simplifying the meta-learning process without needing second-order derivatives. However, thorough studies across various EEG paradigms and datasets are necessary to fully assess the effectiveness of meta-learning techniques, including Reptile, for EEG classification tasks.
  (b) *Feature transfer*: The incorporation of feature transfer constitutes a fundamental element within Implicit Transfer Learning, facilitating the transfer of knowledge from a source domain to a target domain without direct emphasis on the target task. Embedding Learning (EL) emerges as a pivotal technique in this context, overseeing the transformation of samples into a lower-dimensional space where similar samples converge while dissimilar samples differentiate (Willmore, 2013; Vedaldi et al., 2014). EL mitigates the need for an extensive array of training instances by cultivating a more condensed hypothesis space, drawing upon prior knowledge and domain-specific data. The process of embedding captures inherent patterns within the data, thereby facilitating efficacious knowledge transfer. In the domain of Implicit Transfer Learning, approaches such as Feature Transfer, particularly through Embedding Learning, enhance the capabilities of Machine Learning models, enabling adept adaptation to novel tasks even in scenarios with limited labeled samples.
    (1) *Representation learning* To represents any data for Machine Learning algorithms, especially Neural Networks to understand is referred as Embeddings and therefore there is another name to it called Embedding Learning (EL). As Willmore (2013) and Vedaldi et al. (2014) embeds each sample $x_{i}\in X\subseteq {\mathbb{R}}^{d}$ to a lower-dimensional *z*_*i*_ ∈ $Z\subseteq {\mathbb{R}}^{m}$, such that similar samples are close together while dissimilar samples can be more easily differentiated. In this lower-dimensional Z, one can then construct a smaller hypothesis space $\tilde{H}$, which subsequently requires fewer training samples. The embedding function is mainly learned from prior knowledge and can use task-specific information from *D*_train_. Table 10 summarizes the development that happened in the area of Feature transfer for Implicit Transfer Learning.
      EL is the most common technique in transfer learning, where three different embedding styles are popular: task-specific, task-invariant, and hybrid, a combination of task-specific and task-invariant.
      Suh and Kim (2021) proposed the adoption of common spatial patterns through manifold learning. This method is efficiently able to represent 2D features through Riemannian geometry. Hence, when evaluated on cross-subject and session tasks yields acceptable results for 80 % of subjects without losing the overall accuracy,
      Yu et al. (2022) proposed the learning of embedding through decomposition using a DeepSeparator model, which is a sequence-to-sequence model. This inherent separation strategy effectively denoizes and identifies the EEG signal's artifacts. Both the encoder and decoder are composed of inception modules.
      Thiyagarajan et al. (2017) applied triplet loss to the TUH dataset Obeid and Picone (2016) for clustering. The author proposes a CNN network optimized using triplet loss along the euclidean distance. Using the embedding, the authors obtained k-clusters using the elbow method. Visualization through TSNE made it evident that this approach yields good embedding.
    (2) *Multi task learning (MTL)* Multitask learning (Caruana, 1997; Zhang and Yang, 2021) learns numerous related tasks simultaneously using both general and task-specific data. According to Autthasan et al. (2021), this is the first and only study to employ a single EEG dataset for two separate objectives, such as reconstruction and classification, to provide a regularization impact in a dataset of such a low size such for Motory Image classification. MTL is, therefore, a natural choice for FSL as it can generalize well across tasks.
  In the realm of BCIs, the hybrid approaches proposed recently, namely the Double Stage Transfer Learning (DSTL; Gao et al., 2023a) and the Multi-layer Transfer Learning Algorithm based on Improved Common Spatial Patterns (MTICSP; Gao et al., 2023b), garnered attention for their innovative methodologies and promising results. DSTL Gao et al. (2023a) tackled the challenge of limited EEG signal quantities in BCIs by employing a double-stage transfer learning strategy. It first utilized Euclidean alignment for aligning EEG trials from different subjects and subsequently reweighted aligned trials based on the distance between covariance matrices of the source and target domains. After extracting spatial features using Common Spatial Patterns (CSP), transfer component analysis (TCA) was applied to further reduce domain differences. Experimental results on two public datasets [BCIC IV—Dataset 1 (Blankertz et al., 2007) and BCIC IV—Dataset—2a (Brunner et al., 2008)], employing multi-source to single-target (MTS) and single-source to single-target (STS) transfer paradigms, demonstrated superior classification accuracy—84.64 and 77.16% in MTS, 73.38 and 68.58% in STS. This indicated the effectiveness of DSTL in mitigating domain differences and outperforming existing state-of-the-art methods in EEG data classification.
  Similarly, MTICSP Gao et al. (2023b) addressed challenges in decoding algorithms for BCIs, particularly focusing on motor imagery tasks. The algorithm first aligned source and target domain data using Target Alignment (TA) to reduce distribution differences between subjects. Subsequently, the mean covariance matrix was re-weighted based on the distance between covariance matrices of each trial in the source and target domains. An improved Common Spatial Patterns (CSP) technique, introducing a regularization coefficient, was then employed to further reduce differences between source and target domains and extract features effectively. Finally, Joint Distribution Adaptation (JDA) aligned feature blocks from the source and target domains. Experimental evaluations on two public datasets using MTS and STS paradigms demonstrated the efficacy of MTICSP, achieving classification accuracies of 80.21 and 77.58% in MTS, and 80.10 and 73.91% in STS for 5-person and 9-person datasets, respectively. These results underscored the superiority of MTICSP over existing algorithms, showcasing its potential in combining transfer learning with motor imagination tasks in BCIs.

**Table 9:** Explicit Transfer Learning with non-parametric alignment and adversarial learning categorized by column Explicit Learning (EL) type.

**References**	**EEG par**	**TL char**	**EL type**	**Model**	**Datasets**	**Results**	**Main contribution**
Azab et al. (2019)	MI	CS	NPA	LR+CSP	19 subjects' data; BCIC-IV-2a; BCIC-III-4a	70.3%; 75%; 75%	Utilizes KL divergence for measuring similarity in transfer learning.
Giles et al. (2019)	MI	CS	NPA	CSP+LDA	BCIC-IV-2a	77%	Uses Jensen Shannon ratio for measuring similarity and adopts rule adaptation TL.
Adair et al. (2017)	P300	CS	NPA	Bayesian LDA	8 participants' data	62.50%	Ensembles learning of generic information in transfer learning.
Wei et al. (2018)	SSVEP	CS	NPA	Cluster	8 subjects' data	-	Performs variability assessment using Fisher's discriminant ratios in transfer learning.
Hossain et al. (2018b)	MI	CS	NPA	LDA	BCIC-IV-2a	76%	Proposes selective informative expected decision boundary in transfer learning.
Sybeldon et al. (2017)	SSVEP	CS	NPA	LDA	10 healthy subjects	-	Ensembles learning and similarity measurement with mutual information in transfer learning.
Zhang et al. (2020a)	MI	CS	NPA	CNN	BCIC-IV-2b	-	Proposes instance TL based p-hash in transfer learning.
Jin et al. (2018)	MI	CS	NPA	Discriminant Analysis	6 subjects	61%	Adaptive Selective CSP
Özdenizci et al. (2019b)	MI	CSS	AL	DANN - cVAE	GigaScience - MI	-	Adds an adversarial network to cVAE and trains cVAE and classifier separately.
Özdenizci et al. (2020)	MI	CSS	AL	DANN - cVAE	BCI2000	69.8%	Devises DANN by exploiting various CNN-based architectures as their feature extractor.
Zhao et al. (2020)	MI	CT	AL	DANN - CNN	BCIC-IV	83.98%	Adds centre loss for the target to minimize intra-class compactness and maximize inter-class separability.
Tang and Zhang (2020)	MI	CT	AL	DANN - CNN	BCIC-IV	74.55%	Feeds the output of a classifier into a domain discriminator.
Jeon et al. (2019)	MI	CT	AL	DANN - CNN	HGD	92.50%	Selects the source based on resting-state EEG signals.
Wei et al. (2020)	RSVP	CSS	AL	DANN - CNN	11 subjects	-	Selects sources based on the ranking of performances in subject-specific classifiers.
Wang et al. (2021)	ER	CSS	AL	SPD Matrix + CNN	Dreamer; Deap	75.44%	Selects sources based on a ranking of performances in subject-specific classifiers and devises centroid alignment loss.
Nasiri and Clifford (2020)	Sleep	CS	AL	DANN - CNN	SHHS; P18C	84%; 85%	Estimates attention maps using channel-wise domain discriminators.
Ma et al. (2019)	Drowsy	CS	AL	DANN - ResNet	SEED	-	Trains additional parameters capturing subject-specific features.

Ref, Reference; EEG Par, EEG Paradigm; TL Char, TL Characteristics; CS, Cross Session; CD, Cross-Device; CT, Cross Task; CSS, Cross Session and Subject; EL, Explicit Learning; NPA, Non-Parametric Alignment; AL, Adversarial Learning.

**Table 10:** Implicit Transfer Learning via feature transfer and parameter transfer.

**References**	**EEG par**	**TL char**	**IL type**	**Model**	**Datasets**	**Results**	**Main contribution**
Jeon et al. (2019)	MI	CS	RL	CNN	BCIC-IV-2a	-	DA with PSD
Pal et al. (2019)	MI	CS	MultiT	Linear SVM	BCIC-III-IVa	75.80%	Many objective optimization
Hossain et al. (2018a)	MI	CS	RL	LDA	BCIC-IV-2a	67.70%	Informative TL with AL
Yin et al. (2017)	ER	CS	RL	Least-squares SVM	DEAP dataset	78%	Transfer recursive feature elimination
Dai et al. (2019)	MI	CD	MultiT	SVM	BCIC-III-Iva 5 subjects	81.6% 76%	Multiple kernel boosting
Nakanishi et al. (2019)	SSVEP	CD	RL	TRCA	10 subjects	-	Spatial filtering transfer for latent features
Salami et al. (2017)	MI	CSS	RL	SVM	BCIC-IV-2a	89.30%	Fuzzy TL based on generalized hidden-mapping RR
Rodrigues et al. (2018)	Multitask	CD	MultiT	-	8 publicly available BCI datasets	-	Geometrical transformations on Riemannian Procrustes analysis
Waytowich et al. (2016)	ERP	CD	RL	MDRM	15 subjects	62%	Spectral transfer using information geometry
Gaur et al. (2019)	MI	CS	RL	LDA	BCIC-IV-2a	-	Tangent space-based TL
Dai et al. (2018)	MI	CSS	RL	SVM	BCIC-III-IVa	81.14%	Transfer kernel CSP
Zanini et al. (2017)	MI/ ERP	CS	RL	Minimum distance mean & Bayes classifier	BCIC-IV-2a Brain Invaders experiment	-	Affine transform
Jayaram et al. (2016)	MI	CSS	MultiT	RR +SVM	10 healthy subjects an ALS subject data	85%	Multitask learning
Yair et al. (2019)	MI	CT	ZT	Linear SVM	BCIC-IV-2a	-	Zero Shot: Domain adaptation parallel transport on the cone manifold of SPD
Chiang et al. (2019)	SSVEP	CS	PT	-	BCIC-IV-2a	82.10%	Fine Tuning: Least-squares transformation
Xu et al. (2019)	MI	CSS	FT	CNN	BCIC-IV-2b	74.20%	Fine Tuning: Based on VGG16
Sakhavi and Guan (2017)	MI	CS	FT	-	BCIC-IV-2a	69.71%	Fine Tuning: Based on pre-trained network
Behncke et al. (2018)	ErrPs	CT	FT	CNN	Proprietary 15 epilepsy patients	81.50%	Fine Tuning: Based on pre-trained network
Pati et al. (2023)	MI	Cross Subject	MetaT	Meta-learning with optimization-based algorithms; uses EEGNet architecture	BCI Competition IV 2a	55.56% (0 shots)63.13% (10 shots)	Utilized optimization-based meta-learning to achieve better initialization for rapid adaptation to new subjects with limited data, demonstrating superior performance over transfer learning.
Li et al. (2021a)	MI	Cross Session	MetaT	Model-Agnostic Meta-Learning (MAML) with convolutional layers	Physionet EEG motor imagery dataset	60-80%	Applied MAML to train EEG BCI decoders, enabling rapid generalization to new users and sessions through few-shot learning and gradient-based optimization.
Duan et al. (2020)	MI, Emotion	Cross Subject	MetaT	Meta UPdate Strategy (MUPS-EEG)	BCI IV-2a, DEAP	76.3% (BCI IV-2a)67.2% (DEAP)	Proposed MUPS-EEG, a meta-update strategy leveraging meta-learning to extract versatile features and perform fast adaptation, retaining knowledge across subjects.
Jaiswal et al. (2023)	Emotion	Cross Subject	MetaT	Meta-learning-based augmented domain adaptation (MeLaDA)	SEED	86.4%	Developed MeLaDA, incorporating meta-learning and adversarial learning with a distributional shift controller for efficient domain adaptation, achieving a 13% improvement in accuracy compared to the baseline.
Ng and Guan (2024)	MI, Inner Speech	Cross Subject	MetaT	Few/zero-shot subject-independent meta-learning framework	Multiple EEG datasets	88.70% (Binary MI)	Developed a meta-learning framework that uses known subjects to prime the model for faster adaptation with only target samples during fine-tuning, enhancing cross-subject generalization without significant computational overhead.
An et al. (2020a)	MI	CS	MetaT	CNN + Attention	BCIC-IV-2b	74.60%	Meta-Learning: Estimates relation scores amongst source & query
Han et al. (2024)	MI	Cross Subject	MetaT	META-EEG with gradient-based meta-learning and intermittent freezing strategy	Multiple public EEG datasets	Outperformed baseline by 12%	META-EEG combines gradient-based meta-learning with an intermittent freezing strategy to efficiently learn feature representations. This framework showed robust performance and generalizability using a leave-one-subject-out cross-validation (LOOCV) training strategy, enhancing zero-calibration MI-BCI systems.

EEG Par, EEG Paradigm; TL Char, TL Characteristic; CS, Cross Subject; CD, Cross-Device; CT, Cross Task; CSS, Cross Session and Subject; IL Type, Implicit Learning Type; RL, Representation Learning; MultiT, Multi Task Transfer; ZT, Zero Shot Transfer; MetaT, Meta Learning; FT, Fine Tuning.

### 3.3 Self supervised learning (SSL)

Transfer Learning serves as a fundamental cornerstone in our dynamically progressing world, playing a pivotal role in fostering technological and human advancements. The efficacy of Transfer Learning is intricately tied to the quality of the pre-trained model utilized, as it mitigates the necessity for copious amounts of data during the subsequent fine tuning process. This imperative has spurred the emergence of Self-supervised Learning (SSL), leveraging expansive unlabeled datasets to cultivate a comprehensive feature representation within the pre-trained model. Consequently, this optimization streamlines and enhances the fine tuning procedure for subsequent tasks characterized by limited labeled data availability.

SSL employs similarity functions, such as Contrastive Learning (CL; Le-Khac et al., 2020), or Generative Models (Kostas et al., 2021; Li et al., 2022; Ho and Armanfard, 2023), to extract meaningful data representations from unlabeled datasets. A salient attribute of SSL lies in the introduction of a distinct task, the tretext task. Diverging from the original task, the pretext task assumes a pivotal role in effectively acquiring and transferring knowledge to downstream applications. This nuanced approach marks a notable dimension within the realm of Self-supervised Learning.

As discussed by Rafiei et al. (2022), there exist two primary techniques for pretext training: the “Optimal Augmentation Technique” and “Contrastive, Generative, or Hybrid EEG Recognition.” Notably, for EEG classification challenges, the Contrastive approach coupled with Generative Models has demonstrated efficacy on extensive datasets such as the Temple University Hospital (TUH) dataset for Sleep Staging (Obeid and Picone, 2016).

1. *Optimal Augmentation technique:* Self-supervised learning necessitates a substantial pool of unlabeled data to achieve effectiveness. Nonetheless, acquiring such data presents challenges in terms of collection and expense. Researchers have explored diverse data augmentation techniques to mitigate these challenges to generate synthetic data suitable for self-supervised learning scenarios. For instance, Mohsenvand et al. (2021) introduced multiple self-supervised algorithms and augmentation strategies, including mixup techniques, to enhance the accuracy and sample efficiency of subsequent EEG classification tasks.
2. *Contrastive EEG recognition:* Contrastive learning, a prominent self-supervised paradigm, involves learning data representations by contrasting positive and negative instances. Recent studies have delved into the application of contrastive learning within the domain of EEG signal recognition. BENDR (Kostas et al., 2021), for instance, harnessed transformers and a contrastive self-supervised learning framework to glean insights from extensive EEG data. Moreover, Li et al. (2021b) introduced a self-supervised model tailored for EEG signal representation learning, utilizing aggregate statistics from the dataset to discern patterns linked to different sleep stages.
3. *Generative EEG recognition:* Generative models, another prevalent self-supervised technique, strive to produce synthetic data resembling real data distributions. In the context of EEG signal recognition, recent investigations have explored the application of generative models. Notably, Peng et al. (2021) proposed a Self-weighted, Semi-supervised Classification (SWSC) model capable of emotion recognition from EEG signals. The SWSC model incorporates a self-weighted component that assigns weights to features based on relevance across diverse emotion recognition scenarios, leveraging combinations of labeled and unlabeled data.
4. *Hybrid EEG recognition:* Hybrid models amalgamate multiple self-supervised learning approaches to yield highly robust and generalizable representations. An example of such a hybrid approach is the Self-Supervised Graph Neural Networks method proposed by Zhang et al. (2021c), which harnesses graph neural networks in a self-supervised manner to enhance seizure analysis utilizing EEG signals.

Banville et al. (2019) introduced two methods, relative positioning and temporal shuffling for SSL, which helps to learn a rich representation of EEG signals. A simple CNN-based network was used and trained in a Siamese Network (Dong and Shen, 2018) style to learn embedding. The learnt embedding are used to classify the SleepEDF (Goldberger et al., 2000), MASS (O'reilly et al., 2014) dataset, showcasing FSL capablilities with just 10 labeled samples, although it is still away from matching performance of fully supervised learning.

Kostas et al. (2021) conducted a study in which they built upon the work of Banville et al. (2019), who had introduced the SSL approach for EEG signal processing. Kostas et al. (2021) extended this work by incorporating transformer networks and named the model BErt-inspired Neural Data Representations (BENDR). The model had been trained on the TUEG (Obeid and Picone, 2016) dataset and fine-tuned on P300 (Goldberger et al., 2000), MMI (Goldberger et al., 2000), and BCIC (Schirrmeister et al., 2017) datasets. However, only the P300 dataset had significantly improved using this strategy, indicating that SSL had effectively labeled data without compromising accuracy. Kostas et al. (2021) had adopted an approach used in automatic speech recognition, leveraging a self-supervised training objective to learn compressed representations of raw EEG data. The adapted model had successfully handled different hardware, subjects, and tasks, demonstrating versatility. Moreover, the internal representations and the model's architecture had been fine-tuned for various downstream BCI and EEG classification tasks, outperforming previous research in self-supervised sleep stage classification.

Automated seizure detection and classification from EEG has significantly enhanced seizure diagnosis and treatment (Tang et al., 2022). However, prior studies in this area have yet to adequately address several modeling challenges, including (1) representation of non-Euclidean data structure in EEGs, (2) accurate classification of rare seizure types, and (3) needing a quantitative interpretability approach for localizing seizures. This study addresses these issues by (1) using a Graph Neural Network (GNN) to capture the spatiotemporal dependencies in EEGs, (2) proposing two EEG graph structures that capture electrode geometry or dynamic brain connectivity, (3) introducing a quantitative model interpretability a method that predicts preprocessed signals for the next period to further improve model performance, particularly for rare seizure types.

You et al. (2022) introduces SleepGAN, a novel approach that combined Generative Adversarial Networks (GANs) with few-shot learning algorithms to improve sleep staging classification performance. SleepGAN generated synthetic EEG signals and augmented the sleep stage classification training dataset. It showcased good gains in classification metrics on the SleepEDF dataset, even though it posed challenges in verifying if the generated signals were near the real world and could be used on unseen data.

An et al. (2020b) innovatively proposed an approach to address the challenges of few-shot learning in the context of EEG-based motor imagery (MI) classification. The authors devised a two-way few-shot classification network that incorporated attention mechanisms, thereby accentuating pertinent features within the support data. This architectural augmentation aimed to enhance the model's generalization performance when confronted with previously unseen subjects. The outcomes of the proposed approach were substantiated through evaluations conducted on the BCI Competition IV 2b dataset, showcasing a substantial improvement in classification accuracy. The paper's notable contributions encompassed the introduction of a pioneering few-shot attention technique, the integration of 1D Convolutional Neural Networks (CNNs), and the application of few-shot learning principles in the realm of EEG-based MI classification. However, the authors conscientiously acknowledged and addressed considerations pertaining to data requirements and the applicability of the proposed approach in online learning scenarios.

In a manner akin to the BENDR framework (Kostas et al., 2021), BrainBERT, as introduced by Wang et al. (2023), involves the acquisition of EEG signal representations through training on a dataset comprising observations from 10 subjects engaged in diverse activities such as viewing distinct movies. Notably, annotations pertaining to Sentence onset Speech/Non-speech, Volume, and Pitch were applied to the movie clips for the purpose of classification. The resultant representations offer three primary advantages. Firstly, they enhance the accuracy and efficiency of neural decoding, a crucial aspect given that pivotal findings in neuroscience often rely on the performance of linear decoders in specific tasks. Given the common limitation of working with small datasets in neuroscience experiments, a substantial reduction in the requisite data for decoding has significant implications. This is particularly relevant in the context of developing advanced brain-machine interfaces. Secondly, these representations achieve performance enhancement while preserving the interpretability characteristic of linear decoders, even when compared to the complexity of more intricate decoders. This represents a direct enhancement of a widely utilized technique in neuroscience. Finally, by generating task-agnostic embeddings open to subsequent analysis, BrainBERT facilitates novel investigations into cognitive processes. The temporal evolution of representations may uncover mechanisms and dynamics underlying phenomena such as sleep and other alterations in brain states in a fully data-driven manner. To refine the representations constructed by BrainBERT further, future endeavors aim to train more expansive variants on continuous 24/7 recordings from numerous subjects.

## 4 Research gaps and future directions

Research in EEG signal classification using FSL has significantly progressed in recent years. However, some open challenges and research gaps still need to be addressed to improve the accuracy and robustness of the models. This section discusses some of these research gaps and open challenges.

### 4.1 Data augmentation

Despite an extensive body of research concerning data augmentation, particularly in the context of EEG signals, there is a notable absence of a standardized evaluation methodology among researchers. A limited number of investigations have addressed challenges associated with data augmentation, specifically addressing issues such as data skewness resulting from class imbalance within control classes. For instance, Freer and Yang (2020) employed the data skew value to quantify the degree of augmentation required for each class. Moreover, as emphasized by Rommel et al. (2021), the augmentation of EEG signals does not guarantee the preservation of the original class after augmentation. Consequently, an approach involving class-by-class augmentation is considered more efficacious and secure. Importantly, there exists no current research that validates the appropriateness of chosen augmentation techniques; instead, reliance is placed on extrinsic evaluations to demonstrate effectiveness. The following research gaps underscores the need for future investigations:

1. *Need for intrinsic evaluation*: While the majority of research works rely on extrinsic evaluation criteria to demonstrate the efficacy of augmentation techniques, a notable gap arises due to the absence of intrinsic methodologies. The assessment of the effectiveness of the generated signal poses challenges, particularly in gauging its fidelity to real-world scenarios. A critical consideration involves ascertaining whether the generated signal maintains constancy in terms of class, session, and subject in comparison to the original signal. In contrast to domains such as computer vision, audio processing, and natural language processing, where visual inspection of generated data is viable, the assessment of EEG data presents unique challenges. Consequently, there is a strong need for intrinsic evaluation. One direction could be, the training of a model without augmentation, followed by the computation of test metrics. Subsequently, augmentation is applied to the test split, and the test metrics are recalculated. The test metrics should exhibit a comparable range if the augmentation technique effectively approximates real-world conditions.
2. *Addressing the Challenge of Limited Labeled Samples in Few-Shot Learning through Class Invariant Data Augmentation*: Drawing upon the insights derived from Section 3.1.2 and the Tables 5–8, which elucidate diverse augmentation methodologies including Geometric Transformations, Noise Injection, Generative Adversarial Networks, and Sliding Window, it becomes apparent that the predominant spectrum of existing data augmentation techniques within the realm of FSL predominantly exhibits subject or session invariance. Consequently, there exists an imperative for further investigation and innovation in data augmentation strategies specifically oriented toward achieving class invariance. The overarching goal is to systematically generate an extensive array of synthetic samples transcending class invariance, thereby empowering FSL models to attain heightened performance and enhanced generalization across classes.
3. *Revolutionizing Automatic Data Augmentation Techniques for FSL:* Traditional data augmentation methods often require manual specification of augmentation parameters and careful consideration of transform choices to preserve signal semantics (Rommel et al., 2021). In the context of Few-Shot Learning (FSL) with limited labeled samples, there is a need to revolutionize automatic data augmentation techniques that can adaptively augment data per class without extensive labeled data or reliance on generative models. The work done by Rommel et al. (2021) is the only work that explores the automatic data augmentation for EEG signal processing. Hence, further exploration and development of novel automatic data augmentation approaches are required to fully harness the potential of data augmentation in FSL for EEG signals.
4. *Elevating Multivariate EEG Signal Augmentation in the FSL Paradigm:* Augmenting multivariate EEG signals presents an open area of work, as most existing data augmentation techniques focus on univariate augmentation such as Geometric Augmentations and Noise Injections discussed in Section 3.1.2 and Tables 5, 6. However, in the context of FSL, considering multivariate dimensions of EEG signals is crucial. Current approaches augment individual channels independently, overlooking the intricate interdependencies among them. Hence, it is necessary to develop channel-wise augmentation techniques in the frequency domain and convert them into volumetric representations. Additionally, extending spectral-domain augmentation methods, such as SpecAugment, to three-dimensional volumes with multiple channels would capture inter-channel relationships and significantly enhance the generalization capabilities of FSL models on multivariate EEG data (Park et al., 2019).

### 4.2 Transfer Learning

1. *Cross-task learning:* The cross-task learning paradigm remains a significant challenge in transfer learning for EEG signal processing. Label alignment addresses this challenge, which aims to transfer classes from one task to another. However, the effectiveness of label alignment approaches can vary, highlighting the necessity for a valid Euclidean alignment (Eisenhart, 2016). Despite advancements, this remains an open problem in the context of transfer learning for EEG signal processing, emphasizing the need for further exploration and development of novel approaches to enhance cross-task learning in this domain.
2. *Cross-device transfer:* The transfer of learned models from one EEG sensor to another poses significant challenges due to the availability of different EEG sensor vendors with varying device specifications. This variability makes it difficult to transfer data, even for similar tasks. As a result, existing research in transfer learning for EEG signal processing, such as studies by Koelstra et al. (2011) and Cimtay and Ekmekcioglu (2020), has primarily focused on specific datasets collected from particular EEG sensors. These studies have explored the transfer ability of models within datasets collected from different devices for the same task. However, there remains a notable research gap in addressing the challenges of transferring models across different EEG sensor vendors, which requires further investigation and development of robust transfer learning techniques in EEG signal processing.
3. *Transferability of domain validity:* The prevailing body of research predominantly substantiates the plausibility of Cross-Session and Cross-Subject transfers. However, the question pertaining to the attainability of Cross-Task transfer remains a subject of unresolved inquiry. Recent advancements, exemplified by the BENDR framework (Kostas et al., 2021), have delved into the realm of cross-task transfers, employing pretext learning on extensive Sleep Staging data and subsequent fine tuning on downstream tasks such as Motory Image (MI) or Event-Related Potential (ERP) tasks. Despite these endeavors, it is noteworthy that BENDR (Kostas et al., 2021) has yet to manifest performance levels comparable to fully supervised equivalents. Furthermore, research conducted by Lan et al. (2018) elucidates that tasks originating from the same domain but captured by disparate sensors, as evidenced by the transition from DEAP (Koelstra et al., 2011) to SEED (Miller et al., 2014) or vice versa, can indeed be effectively transferred. These divergent findings prompt a pivotal inquiry: does the scope of transferability extend solely to tasks that are identical or closely related in nature?
4. *Meta-learning techniques:* Despite recent advancements in meta-learning, there remain challenges and uncertainties regarding their effectiveness for EEG classification tasks. For instance, Nichol et al. (2018) posits that recent advancements in meta-learning, such as Reptile algorithms, may not significantly enhance performance in EEG tasks, particularly motor imagery classification. The Reptile algorithm is a first-order meta-learning approach designed for fast adaptation to new tasks by optimizing for a good initialization with gradient descent steps on randomly sampled tasks. Comprehensive studies across various EEG paradigms and datasets are essential to thoroughly evaluate the efficacy of meta-learning techniques, including Reptile, for EEG classification tasks.

### 4.3 Self supervised learning

The lack of massive datasets in EEG signal processing has posed challenges in developing foundational models using self-supervised learning. While inspiring works like BENDR (Kostas et al., 2021) have explored the potential of self-supervised learning, searching for a promising foundational model for downstream tasks still needs to be more conclusive. The absence of extensive labeled datasets hinders the exploration of self-supervised learning techniques and their effectiveness in the context of EEG signal processing. Addressing this challenge and developing robust foundational models that leverage self-supervised learning for EEG signals require further research and efforts in data collection and annotation. SSL has been successful in image, audio, and language processing tasks, and it can similarly benefit EEG signal processing by providing a foundation model. However, some challenges prevent achieving state-of-the-art performance, such as limited data availability or smaller architecture. Kostas et al. (2021) encountered analogous challenges in their study, conducting pretext learning on the Sleep Staging dataset—TUEG (Obeid and Picone, 2016), which currently stands as the only expansive open EEG dataset available.

### 4.4 Practical implications and real-world spplications

Although theoretical advancements in Few-Shot Learning (FSL) have shown promise, practical implementation poses additional challenges. This section explores the practical implications, challenges, and provides case studies demonstrating the application of FSL techniques in real-world EEG scenarios.

#### 4.4.1 Practical implications

1. **Low signal-to-noise ratio**: EEG signals are often contaminated with noise from various sources, including muscle activity and environmental interference. Effective preprocessing and noise reduction techniques are crucial.
2. **High dimensionality**: EEG data is high-dimensional, making it computationally intensive to process. Dimensionality reduction techniques like PCA or t-SNE can be used to mitigate this issue.
3. **Inter-individual variability**: EEG features can vary significantly between individuals due to differences in brain anatomy and physiology. Personalized models or domain adaptation techniques are essential to address this variability.
4. **Real-time processing**: For applications like BCIs, real-time processing is critical. Implementing efficient algorithms and optimizing code for speed are necessary to meet real-time constraints.

#### 4.4.2 Case studies

##### 4.4.2.1 Case study 1: epileptic seizure detection (Tang et al., 2022)

- **Scenario**: Detecting epileptic seizures in patients using EEG data.
- **Data collection**: EEG data from clinical visits.
- **FSL technique**: A Graph Neural Network (GNN) combined with FSL algorithms.
- **Challenges**:
  - High inter-patient variability.
  - Noise in clinical EEG recordings.
  - Accurate classification of rare seizure types.
- **Solutions**:
  - **Data Augmentation (DA)**: Employed various data augmentation techniques to artificially increase the size of the training dataset, helping the model learn more robust features.
  - **Transfer Learning (TL)**: Used pre-trained models on large EEG datasets and fine-tuned them on the specific dataset for seizure detection.
  - **Graph Neural Networks (GNN)**: Utilized GNNs to capture spatiotemporal dependencies in EEG signals, which are crucial for accurate seizure detection.
  - **Model interpretability**: Introduced a quantitative model interpretability method to predict preprocessed signals for the next period, improving the model's performance, particularly for rare seizure types.
- **Outcome**: Achieved significant improvements in classification accuracy, particularly for rare seizure types.

##### 4.4.2.2 Case study 2: cognitive load monitoring in educational settings (Kuanar et al., 2018)

- **Scenario**: Monitoring students' cognitive load during learning activities using EEG.
- **Data collection**: EEG data from students performing cognitive tasks.
- **FSL technique**: Deep Recurrent Neural Networks (RNNs) combined with FSL algorithms.
- **Challenges**:
  - Variability in cognitive load patterns.
  - Real-time analysis requirements.
- **Solutions**:
  - **Data Augmentation (DA)**: Applied various DA techniques to create synthetic data, enhancing the training process.
  - **Self-Supervised Learning (SSL)**: Leveraged SSL techniques to pre-train the model on large unlabeled EEG datasets, followed by fine-tuning on the specific task of cognitive load monitoring.
  - **Deep Recurrent Neural Networks (RNNs)**: Used RNNs to capture the temporal dynamics of EEG signals, which are crucial for monitoring cognitive load.
  - **Real-time processing algorithms**: Implemented efficient real-time processing algorithms to ensure immediate feedback during learning activities.
- **Outcome**: Achieved high accuracy in cognitive load monitoring with limited labeled data.

These case studies illustrate the practical challenges of implementing FSL techniques in real-world EEG applications. They also highlight the importance of developing robust preprocessing methods, effective data augmentation strategies, and efficient real-time processing algorithms.

## 5 Best practices for Few-Shot EEG signal classification

**Figure 7:**
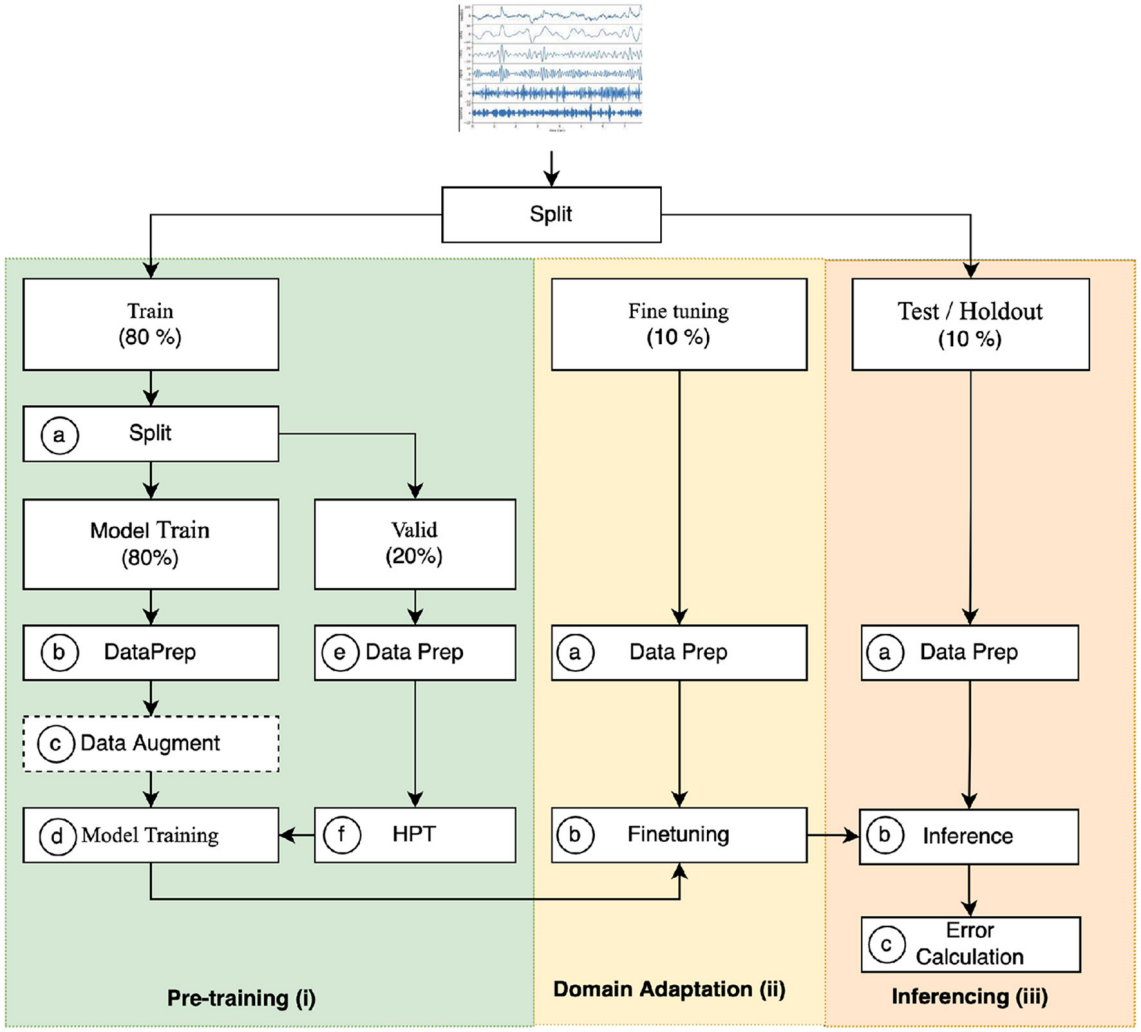
Ideal strategy for employing Few-Shot learning. HPT, Hyperparameter Tuning.

Few-shot learning (FSL) has emerged as a pivotal research frontier in EEG signal classification, addressing the challenging task of classifying brain signals with limited labeled data. This survey delves into an extensive exploration of various paradigms within EEG signal classification and investigates how they can be harnessed to enhance FSL methodologies. This section presents a comprehensive set of best practices from empirical findings and methodological insights. These best practices encompass data augmentation techniques, robust validation strategies, transfer learning considerations, and model adaptation nuances. By adhering to these guidelines, researchers and practitioners can navigate the intricate landscape of FSL for EEG signal classification, ultimately fostering advancements in this critical field. Transfer learning and self-supervised learning are two techniques that show the potential for achieving zero- or few-shot learning. Figure 7 illustrates three phases an FSL algorithm should undergo to study the approach's effectiveness, as outlined below.

When developing a model and assessing the effectiveness of any modeling technique or methodology, it is crucial to implement an unbiased split to prevent data leakage during evaluation. Therefore, an 80–20 split is commonly used as a general guideline in most machine-learning problems. Train split should exclusively use this data split for the modeling process in the pre-training phase; fine-tuning split similarly exclusively uses this data split for fine-tuning the pre-trained model. Use the testing split, which should only be used for reporting classification metrics. One can avoid the fine-tuning split if fully supervised learning is employed without any fine-tuning considerations. For imbalanced data, stratified sampling with class distribution is recommended. Similarly, consider subjects/sessions as strata in Leave-One-Subject-Out (LOSO) style if evaluating cross-subject or session. For instance, split N subjects into three splits: designate N-3 subjects for training, the N-2th subject for fine-tuning, the N-1th subject for fine-tuning if doing domain transfer, and the Nth subject for test evaluation. Similarly, for cross-session evaluation, allocate m-3 sessions for each subject to training, the m-2th session for validation, the m-1th subject for fine-tuning and the mth session for test evaluation, assuming each subject has m sessions. Once data is split carefully, it is safe to begin pre-training, fine-tuning, and finally, inferencing to report the errors.

1. *Pre-training:* Pre-training is an initial stage in developing a model that understands rich feature representations for downstream tasks. For Few-Shot Learning (FSL) problems, using a technique that does not allow fine-tuning of the pre-trained model is not advisable unless a model emerges with great zero-shot transfer capabilities. The following steps outline the ideal training protocol:
  (a) *Model training and validation split:* While training any model, there is a need for Hyperparameter Tuning (HPT). Therefore, it is advisable to use a separate split as validation.
  (b) *Data preparation:* Any EEG signal processing starts with artifact removal and baseline calibration concerning the sensor. Also, normalize the data either using standardization or any other technique.
  (c) *Data augmentation:* Most TL research uses DA. However, they do not ablate DA but have some positive impact while pre-training, and therefore, it is suggested to augment the training data to increase the number of samples to have a robust pre-trained model. Begin with the Geometric Transformations and Noise Injection as shown in Table 4 in Section 3.1.2 and apply class by class to balance the class distribution for training (Freer and Yang, 2020) until there is a need not use class labels as in SSL. While implementing DA in the Deep Learning setup, introduce DA augmentation during the sampling process so that each batch sees different augmented samples rather than the same in every epoch.
  (d) *Model training:* Train the model parameters and the hyperparameters using a validation split to develop an optimal training configuration that yields the best model on the validation split by iteratively performing Steps *eandf*.
  (e) *Data preparation:* During validation for hyperparameter tuning, use the same preprocessing logic and parameters like mean and standard deviation computed for standardization and artifact removal.
  (f) *Hyperparameter tuning:* Usually, it is required to tune the training and model hyperparameters, such as learning rate, optimizer, batch size, and number of epochs. For this, a manual process or automatic algorithms, which are usually iterative, can be employed.
  (g) *Diverse data sources:* Use multiple EEG datasets representing various populations, experimental conditions, and hardware setups. This ensures that FSL techniques are tested across a broad spectrum of scenarios.
  (h) *Dataset description:* Provide detailed descriptions of each dataset used, including participant demographics, recording conditions, and hardware specifications.
2. *Domain adaptation:* It is advisable to begin any modeling without the consideration of domain adaptation. If model cross-subject or session performance is poor during testing, consider transferring the subject or session using domain adaptation techniques.
  (a) *Data preparation:* Once the pre-trained model is available, use the same preprocessing logic to prepare the fine-tuning split.
  (b) *Finetuning:* Adjust the parameters of the target domain through fine-tuning or align the data using the designated fine-tuning split. Commencing the process involves selectively fine-tuning segments of the pretrained neural network to facilitate the transfer of knowledge pertaining to EEG analysis (Zhao et al., 2019; Zhang et al., 2021b). In instances involving a novel subject, (Zhao et al., 2019; Zhang et al., 2021b) specifically fine-tuned solely the parameters associated with fully-connected layers while maintaining the immutability of preceding layers. In cases where model overfitting occurs due to a lack of samples, it is advisable to prioritize domain alignment over fine-tuning. The Riemannian Alignment (RA) method is recommended for aligning EEG covariance matrices from distinct subjects within the Riemannian space, while an alternative approach involves utilizing the Euclidean space (EA). Research conducted by He and Wu (2019) illustrates the superiority of EA over RA alignment in terms of efficiency. Notably, EA necessitates a dataset size ranging between 20 and 50 examples, whereas RA alignment can be achieved with a smaller dataset size of 3–5 examples (Chiang et al., 2019). Consequently, initiating the process with EA-based domain adaptation is advisable when dealing with a dataset comprising approximately 20 samples for subject transfer. Conversely, in scenarios where the availability of new subject samples is limited, preference should be given to RA-based techniques.
  (c) *Detailed metrics:* Evaluate model performance using a variety of metrics, such as accuracy, sensitivity, specificity, precision, recall, and F1-score.
  (d) *Statistical analysis:* Conduct statistical analyzes (e.g., ANOVA, *t*-tests) to compare performance across different datasets and conditions. Report *p*-values and effect sizes to highlight significant differences.
3. *Inferencing:* For testing on unseen data, use the holdout split kept at the initial phase and not included in the pre-training or fine-tuning phase.
  (a) *Data preparation:* Again, use the same preprocessing steps and parameters for preprocessing, such as baseline correction, artifact removal, and data normalization.
  (b) *Inferencing:* It is safe to make predictions using the fine-tuned model.
  (c) *Error calculation:* Use test/holdout split predictions to report errors or model selection.
  (d) *Cross-hardware validation:* Test FSL techniques on data collected from different EEG devices to assess their robustness against hardware-induced variability.
  (e) *Noise and artifact management:* Implement preprocessing steps to handle noise and artifacts. Techniques such as filtering, Independent Component Analysis (ICA), and robust data augmentation should be used to enhance signal quality.
  (f) *Real-world scenarios:* Discuss the applicability of FSL techniques in real-world clinical and research settings. Include case studies where these techniques have been successfully deployed.
  (g) *Scalability and adaptability:* Address the scalability of FSL techniques to larger datasets and their adaptability to new, unseen data. Propose solutions for any identified limitations.

By following these best practices, researchers can develop and assess FSL models that are robust, generalizable, and applicable across diverse EEG datasets and conditions, ultimately advancing the field of EEG signal classification.

Table 11 provides a detailed overview of various EEG paradigms, the challenges they face, the state-of-the-art techniques used to address these challenges, and the improvements achieved.

**Table 11:** Overview of state-of-the-art techniques and improvements for EEG paradigms.

**EEG paradigm & dataset**	**Challenges**	**State of the art technique (DA/TL/SSL, etc)**	**Improvements**
Motor imagery (BCI competition IV, dataset 2a)	Inter-subject variability, low signal-to-noise ratio	Data augmentation (geometric transformations, noise injection) Freer and Yang (2020), Transfer learning (Fine-tuning) Zhao et al. (2019)	Increased accuracy from 70% to 85% with DA; TL improved cross-subject performance by 10%
Emotion recognition (DEAP, SEED)	High variability in emotional responses, artifact presence	Self-supervised learning (SSL) Schwabedal et al. (2018), Domain adaptation (He and Wu, 2019)	SSL improved robustness to noise; Domain Adaptation increased cross-session accuracy by 15%
Steady-state visually evoked potentials (SSVEP, proprietary dataset)	Consistency across sessions, individual differences	Transfer learning [adversarial learning (Özdenizci et al., 2019b), Euclidean alignment (He and Wu, 2019)]	Adversarial Learning improved session invariance by 12%; EA reduced data requirement by 50%
Rapid serial visual presentation (RSVP, BCIT)	High variability in cognitive responses, low data availability	Generative Models [GANs (Chang and Jun, 2019), VAEs (Komolovaitė et al., 2022)], Transfer Learning [Parameter Transfer (Zhang et al., 2021b)]	GANs increased data diversity and improved accuracy by 8%; TL enabled better generalization with fewer samples
Sleep staging (Sleep-EDF)	Variability in sleep patterns, limited labeled data	Data augmentation [signal decomposition (Kalaganis et al., 2020), Sliding window (Mousavi et al., 2019)], Self-supervised learning (SSL) Schwabedal et al. (2018)	Sliding Window technique improved model robustness; SSL enhanced performance with limited data

## 6 Guidelines for reporting results for FSL research

To ensure the clarity, reproducibility, and consistency of reporting in Few-Shot Learning (FSL) research, authors must adhere to the following positive guidelines when presenting their results. These guidelines aim to facilitate future researchers in easily validating proposed methods against existing techniques:

1. *Thorough methodological description:* Articulate the proposed methodology precisely, elucidating key details such as the number of trials, samples, classes, preprocessing steps, and hyperparameter configurations at each stage of Data Augmentation (DA), pre-training for Transfer Learning (TL), or Self-Supervised Learning (SSL). This meticulous approach, as advocated by prior research (Fahimi et al., 2019; Freer and Yang, 2020; Kostas et al., 2021), provides a detailed description of the methodology, contributing to enhanced reproducibility.
2. *Assessment approach and baseline benchmarking:* In the introduction of novel techniques, the methodology should establish a rigorous assessment strategy and implement a robust baseline for evaluation. Utilization of conventional metrics such as Accuracy, F1-Score, Precision, and Recall is imperative in the context of EEG signal classification. Furthermore, accounting for class distribution is essential, particularly when dealing with imbalanced datasets, and accuracy reporting should reflect this consideration. The presentation of results on a per-class basis is advocated over reliance on micro, macro, or weighted averages. Substantive attention must be given to ensuring a meaningful baseline comparison, especially in the domains of DA, TL, or SSL studies.
3. *Comparative analyzes with Pertinent Methodologies:* In the domain of Data DA, it is crucial for the methodology to compare the devised techniques against robust and substantive baseline methodologies. As noted by Zhang et al. (2023), the proposed DA technique is compared with other generative models alongside state-of-the-art classification algorithms. Similarly, in the execution of TL or SSL investigations, the methodology should undertake comparisons with alternative TL or SSL techniques, avoiding non-TL/SSL approaches. As reported by Eldele et al. (2023), the four SSL approaches are thoroughly evaluated without amalgamating the results with modeling or any other approaches. The central focus must remain on showcasing the effectiveness of DA, TL, or SSL, whether applied independently or in conjunction with these methodologies.
4. *Data leakage free evaluation strategy*: It is important to conduct complete experiments without any data leakage to ensure that results reported across research are comparable. All work around the dataset should use a similar evaluation strategy, as follows:
  (a) *Data split (train, validation, test/holdout) ratio:*
    (1) *Data augmentation:* Report the split percentage used for training and validating the augmentation technique. Also, be clear about the splitting criteria such random or stratified and respective parameters used. Ensure the test split remains untouched during the augmentation process to maintain unbiased evaluation and augmentation is done using train split only.
    (2) *Transfer learning:* Be specific if training on the validation split or if a separate split is used for domain transfer. Have a separate validation split for transferring the domain. Avoid using the test split during the transfer learning process to accurately assess the model's generalization to unseen data.
    (3) *Self supervised learning:* Be explicit about which split labels are used during the fine tuning process train, valid or any other.
  (b) *Amount of samples used during augmentation and domain transfer:*
    (1) *Data augmentation:* Showcase the performance of the final task while changing the augmented samples in the ratio of 0-20% of training data as presented by Rommel et al. (2021) for each transformation they used. It is also suggested to discuss how each class is augmented if maintaining the same distribution or balancing the class distribution as Freer and Yang (2020) augments the more samples of the minority class to make the dataset balanced.
    (2) *Transfer learning:* If transferring a new domain on a validation split or a separate data split. Most of the work does not layout which split is used for hyperparameter tuning and which is for transfer learning, which poses a question of whether the results presented are prone to data leakage. Also, study how transferring 1 to N samples from the chosen split affects the testing metrics.
    (3) *Self-supervised learning:* When dealing with multiple datasets, it is advisable to be specific about how all datasets are combined and trained for pretext learning. Similar to TL, present the few-shot performances across 0-N samples to showcase how many samples are optimal to finetune the pre-trained model.
  (c) *Hyperparameters used during training:*
    (1) *Data augmentation:* Specify the parameter values used for Geometric transformations like rotation ratio, dropout probability, masking ratio, shifting scale, and the amount of noise added using noise injection. Include model training hyperparameters for learning a generating model, such as the optimizer choice, learning rate, and batch size.
    (2) *Transfer learning:* State model architecture and training parameters with the final values, whether tuned or heuristic-based. Include details like learning rate schedules, weight decay, and the optimizer's choice to ensure reproducibility and transparency.
    (3) *Self-supervised learning:* Provide details on pretext data preparation parameters, pretext model architecture, training hyperparameters, and fine tuning parameters, including specifics on how the pretext model's knowledge is transferred to the downstream task.

By adhering to these comprehensive guidelines, researchers can contribute to the advancement of FSL research through transparent and reproducible findings. A good start on few-shot learning research would be to begin with good experimentation frameworks and libraries available in open source. These frameworks, such as BrainDecode, have been active in integrating state-of-the-art models and data augmentation techniques already (MacInnes et al., 2020). Similarly, for SSL, SelfEEG is a good project to look at, as it is actively being pursued to integrate SSL algorithms for EEG (MedMaxLab, 2021). BENDR is another excellent project that leverages transformers and contrastive self-supervised learning to learn from massive amounts of EEG data (Kostas et al., 2021), it is a good example on how to systematically conduct SSL related research and test robustly across various tasks. In general, TorchEEG aims to integrate all EEG-related modeling, augmentation, transfer learning, and pre-training work under one area (Zhou et al., 2022). Instead of starting from scratch, it is always beneficial to start from such projects (AliasVishnu, 2020; SuperBruceJia, 2020). Adding to the list of useful resources, the MMFewShot library is a comprehensive toolbox that supports multiple tasks in few-shot learning, including classification and detection, and offers strong baselines and state-of-the-art methods (OpenMMLab, 2021). For those specifically interested in applying few-shot learning to EEG data, the repository *Reptile_on_EEG* demonstrates the application of OpenAI's Reptile algorithm for EEG classification using few-shot learning techniques (Bielby, 2021). Another valuable project is the *EEG HELPS FEW SHOT LEARNING*, which utilizes contrastive learning to enhance feature extraction from EEG signals and applies these features to few-shot image tasks (Lu, 2024). Apart from these, Table 12 highlights few projects by category, that can easily be followed due to their code structure and good README.

**Table 12:** Summary of open-source projects for EEG Data Augmentation, transfer learning, self-supervised learning, and frameworks.

**Category**	**Project name with citation**	**Description**
Data Augmentation	EEG-AUGMENTATION-BENCHMARK-2022 (Rommel, 2022)	Systematic comparison of data augmentation techniques for EEG.
	EEG Data Augmentation using Variational Autoencoder (Vasarkar, 2021)	Uses VAE to generate synthetic EEG signals for motor imagery classification.
	DeepEEGDataAugmentation (Freer, 2019)	Methods for data augmentation and EEG data processing using deep learning-based classifiers.
	EEG Data Augmentation (Jacobsen, 2021)	Investigates the role of data augmentation on the TUH EEG Artifact corpus.
	EEG Synthetic Data Generation Using Probabilistic Diffusion Models (Tosato et al., 2022)	Generates synthetic EEG data using denoising diffusion probabilistic models.
	PyTorch-GANSER (Zhang et al., 2023)	A GAN-based method for generating synthetic EEG data.
	EEG-GAN (Neuroidss, 2018)	Uses GANs to generate synthetic EEG data for augmentation.
Transfer Learning	EEG-Transfer-Learning (IMICS-Lab, 2021)	Self-supervised EEG data transfer learning using a deep convolutional neural network.
	EEG-Adapt (Zhang, 2020)	Adaptive transfer learning using deep CNNs for EEG motor imagery classification.
	MS-MDA (VoiceBeer, 2022)	Multi-source domain adaptation for EEG-based cross-subject emotion recognition.
	Cross-subject EEG Emotion Recognition (Ferreira, 2020)	Cross-subject emotion recognition through neural networks.
	Reptile_on_EEG (Bielby, 2021)	Applies OpenAI's Reptile algorithm for EEG classification using few-shot learning techniques.
Self-Supervised Learning	SelfEEG (MedMaxLab, 2021)	Integrates SSL algorithms for EEG.
	BENDR (Kostas et al., 2021)	Leverages transformers and contrastive self-supervised learning for EEG.
	EEG HELPS FEW SHOT LEARNING (Lu, 2024)	Utilizes contrastive learning to enhance feature extraction from EEG signals for few-shot learning tasks.
	Eval SSL SSC (Emadeldeen, 2024)	Evaluation of self-supervised learning techniques for semi-supervised classification of EEG signals.
Frameworks	BrainDecode (MacInnes et al., 2020)	Library for EEG machine learning.
	TorchEEG (Zhou et al., 2022)	Integrated framework for EEG modeling, augmentation, transfer learning, and pre-training.
	MMFewShot (OpenMMLab, 2021)	Comprehensive toolbox for few-shot learning tasks, including EEG classification.

## 7 Conclusion

In conclusion, this systematic review has provided an in-depth and integrated perspective on the application of Few-Shot Learning (FSL) techniques, encompassing Data Augmentation (DA), Transfer Learning (TL), and Self-Supervised Learning (SSL) in the domain of EEG signal processing. Our research distinguishes itself from existing reviews by offering a comprehensive and forward-looking approach, introducing a novel taxonomy that categorizes and organizes these techniques, facilitating a structured understanding of their applicability across a diverse range of EEG paradigms.

The challenges and opportunities discussed in this review not only shed light on the current state of FSL in EEG signal analysis but also lay the foundation for future research directions. Addressing the challenges posed by limited labeled data, inter-subject variability, and the need for robust models, this review propose avenues for further exploration. Future research can actively focus on advancing Transfer Learning strategies to enhance model adaptation to new domains, robustness to out-of-distribution data, and generalization across EEG paradigms. Although the availability of sophisticated open-source frameworks for EEG is limited, this remains an important area of work for the community. Developing such frameworks can provide new researchers with essential tools to bootstrap their research efforts. This paper highlights a few existing frameworks that offer valuable resources for EEG signal processing and analysis. By leveraging these frameworks, researchers can advance the field through transparent, reproducible, and efficient experimentation.

Moreover, the potential of Self-Supervised Learning to reduce data annotation burdens and provide robust EEG signal representations offers exciting prospects for future investigations. Developing SSL techniques tailored for EEG signal processing is a promising area that can actively expand the scope of FSL applications.

Overall, this systematic review not only offers a comprehensive reference for researchers and practitioners in EEG signal processing but also actively inspires and guides future research endeavors and also proposes best practices and guidelines for future research in FSL. The combined potential of Data Augmentation, Transfer Learning, and Self-Supervised Learning, as elucidated in this review, holds the promise of advancing the field of EEG signal analysis, ultimately benefiting applications in neuroscience, human-computer interaction, and clinical diagnosis. The challenges discussed in this review serve as catalysts for pioneering research, actively propelling us toward the development of robust, adaptable, and efficient FSL models, thereby furthering our understanding of the human brain through EEG signal analysis.

## Data availability statement

The datasets presented in this article are not readily available because, there is no data in this study. Requests to access the datasets should be directed to cahuja1992@gmail.com.

## Author contributions

CA: Conceptualization, Formal analysis, Investigation, Methodology, Writing - original draft, Writing - review & editing, Visualization. DS: Supervision, Validation, Writing - review & editing, Visualization.
